# The circadian transcription factor ARNTL2 is regulated by weight-loss interventions in human white adipose tissue and inhibits adipogenesis

**DOI:** 10.1038/s41420-022-01239-3

**Published:** 2022-11-03

**Authors:** Markus Mandl, Hans P. Viertler, Maria Zopoglou, Maria C. Mitterberger-Vogt, Juliane Gasser, Florian M. Hatzmann, Tina Rauchenwald, Marit E. Zwierzina, Monika Mattesich, Alexander K. H. Weiss, Lorenza Mottes, Camille Brucker, Petra Waldegger, Gerhard Pierer, Werner Zwerschke

**Affiliations:** 1 Division of Cell Metabolism and Differentiation Research, Research Institute for Biomedical Aging Research, University of Innsbruck, Innsbruck, Austria; 2 Department of Plastic, Reconstructive and Aesthetic Surgery, Medical University of Innsbruck, Anichstraße 35, A-6020 Innsbruck, Austria; 3 Metabolism and Cellular Senescence Research Group, Research Institute for Biomedical Aging Research, University of Innsbruck, Innsbruck, Austria

**Keywords:** Stem-cell research, Diseases

## Abstract

Misalignment of physiological circadian rhythms promotes obesity which is characterized by white adipose tissue (WAT) expansion. Differentiation of Adipose stem/progenitor cells (ASCs) contributes to WAT increase but the importance of the cellular clock in this process is incompletely understood. In the present study, we reveal the role of the circadian transcription factor Aryl hydrocarbon receptor nuclear translocator-like 2 (ARNTL2) in human ASCs, isolated from subcutaneous (s)WAT samples of patients undergoing routine elective plastic abdominal surgery. We show that circadian synchronization by serum-shock or stimulation with adipogenic stimuli leads to a different expression pattern of ARNTL2 relative to its well-studied paralogue ARNTL1. We demonstrate that *ARNTL2* mRNA is downregulated in ASCs upon weight-loss (WL) whereas ARNTL2 protein is rapidly induced in the course of adipogenic differentiation and highly abundant in adipocytes. ARNTL2 protein is maintained in ASCs cooperatively by mechanistic Target of Rapamycin (mTOR) and Mitogen-activated Protein Kinase (MAPK) signalling pathways while ARNTL2 functions as an inhibitor on both circuits, leading to a feedback mechanism. Consistently, ectopic overexpression of *ARNTL2* repressed adipogenesis by facilitating the degradation of ARNTL1, inhibition of Kruppel-Like Factor 15 (*KLF15*) gene expression and down-regulation of the MAPK-CCAAT/enhancer-binding protein β (C/EBPβ) axis. Western blot analysis of sWAT samples from normal-weight, obese and WL donors revealed that ARNTL2 protein was solely elevated by WL compared to ARNTL1 which underscores unique functions of both transcription factors. In conclusion, our study reveals ARNTL2 to be a WL-regulated inhibitor of adipogenesis which might provide opportunities to develop strategies to ameliorate obesity.

## Introduction

Obesity and its comorbidities are major health issues of the 21st century worldwide [1]. Obese individuals show a phenotype of premature aging and suffer more frequently from age-associated severe diseases [2, 3]. Lifestyle factors such as an altered sleep-wake pattern (e.g., due to shift-work) or high caloric food consumption increase the incidence of obesity [4, 5]. Both cues, “light” and “food intake”, control circadian rhythms [4, 6–8] which represent an adaptive mechanism to coordinate metabolic functions and cellular processes within a predictable 24 h cycle [6]. The central clock in the human brain, the *Suprachiasmatic nucleus* (SCN), is entrained by light whereas peripheral cellular clocks are highly responsive to food intake [6]. Both internal timing systems need to be synchronized by several mechanisms to properly maintain body functions [9].

Obesity is characterized by white adipose tissue (WAT) expansion, which is the outcome of increased adipocyte size (hypertrophy) and/or number (hyperplasia) [10]. New adipocytes are generated by the amplification and differentiation of adipose stem/progenitor cells (ASCs), a process referred to as adipogenesis [10]. Terminal adipocyte differentiation is initiated in response to various extracellular stimuli including insulin and insulin-like growth factor 1 (IGF-1), which activate the Phosphoinositide 3 kinase (PI3K)/Protein kinase B (Akt)/mechanistic Target of Rapamycin (mTOR) signaling pathway and the Rat sarcoma (RAS)/Mitogen-activated protein kinase (MAPK) cascade. These signals converge in the activation of a transcriptional cascade involving among others the early adipogenic transcription factor CCAAT/enhancer-binding protein β (C/EBPβ) and the adipogenic key factor Peroxisome proliferator-activated receptor γ (PPARγ) [11–15]. Endogenous cellular clocks link circadian cues with key regulators of adipogenic differentiation [16, 17]. These molecular time-keeping systems are composed of transcriptional-translational feedback loops and driven by transcription factors belonging to the Per-ARNT-Sim family [18, 19]. The transcription factor Aryl hydrocarbon receptor nuclear translocator-like 1 (ARNTL1; also known as Brain and muscle ARNTL1 (BMAL1), MOP3 or ARNT3 [17]) or its paralog ARNTL2 (also known as BMAL2, MOP9 [20] or ARNT4 [21]) heterodimerizes with Circadian locomotor output cycles kaput (CLOCK) to form a transcriptionally active complex [18, 20, 22]. Subsequently, these heterodimers bind to E-box elements in the promoter of clock-controlled genes (ccgs) and initiate transcription [18]. The ccgs *PER* and *CRY* encode the negative regulators of the circadian clock which in turn inhibit ccgs expression. Thus, together with an auxiliary feed-back loop, an oscillatory gene expression pattern is achieved [18]. Genes controlling circadian rhythms exhibit oscillatory expression in ASCs [23] and alterations of the circadian clock are known to affect adipogenesis [17, 24]. ARNTL1 is involved in the regulation of adipogenesis [17, 25] whereas the role of ARNTL2 in this process is incompletely understood. There is evidence that ARNTL2 can replace ARNTL1 and ameliorate the obese phenotype in ARNTL1 knockout (KO) mice [26] but different functions of both isoforms have also been proposed [27].

Obesity is treated by weight-loss (WL) interventions such as caloric restriction (CR) which can attenuate the aging process in animal models [28, 29] and humans [28, 30–32]. Peripheral circadian clocks have been shown to contribute to this life-prolonging effect in vivo by ARNTL1 dependent and independent mechanisms [29, 33]. Previously our group identified several WL target genes in human ASCs and demonstrated their importance to maintain ASC proliferation and differentiation capacity by preventing cellular senescence [13, 14, 34–36]. This screen also implied ARNTL2 to play a key role in adipose tissue biology [34].

The objective of the present study was to investigate the importance of ARNTL2 in adipogenesis. Our results reveal antagonistic functions of ARNTL1 and ARNTL2 in human ASCs and identify ARNTL2 as an inhibitor of adipogenic differentiation.

## Results

### Weight-loss downregulates *ARNTL2* expression in ASCs

**Fig. 1:**
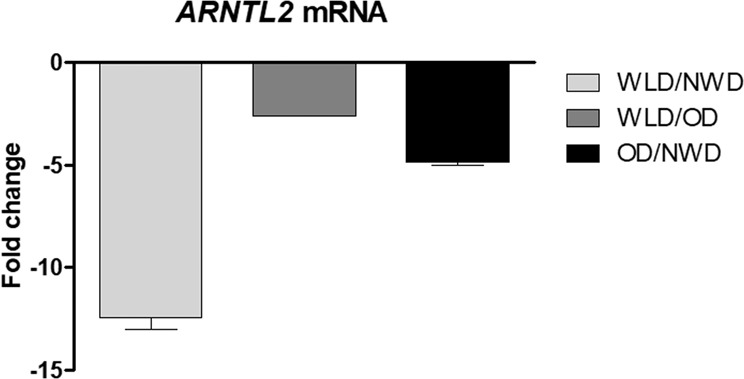
*ARNTL2* expression is downregulated upon weight-loss (WL) in human ASCs. Microarray analysis of freshly isolated ASCs was done as described in Ejaz et al. [34]. Tissue samples were acquired from age-matched normal-weight donors (NWDs; *n* = 3), obese donors (ODs; *n* = 3) and weight-loss donors (WLDs; *n* = 4).

We identified a set of genes in human ASCs which were affected by weight-loss (WL) interventions [34]. ASCs were freshly isolated from subcutaneous white adipose tissue (sWAT) of age and sex matched normal weight donors (NWD), obese donors (OD) and WL donors (WLD) followed by whole genome microarray expression profiling (Affymetrix Chip U133 + 2.0) [34]. Among the WL target genes [34], *ARNTL2* expression was strongly downregulated in ASCs of WLDs (Fig. 1).

### *ARNTL1, ARNTL2* and *C/EBPβ* exert divergent circadian expression patterns upon serum-shock in ASCs

**Fig. 2:**
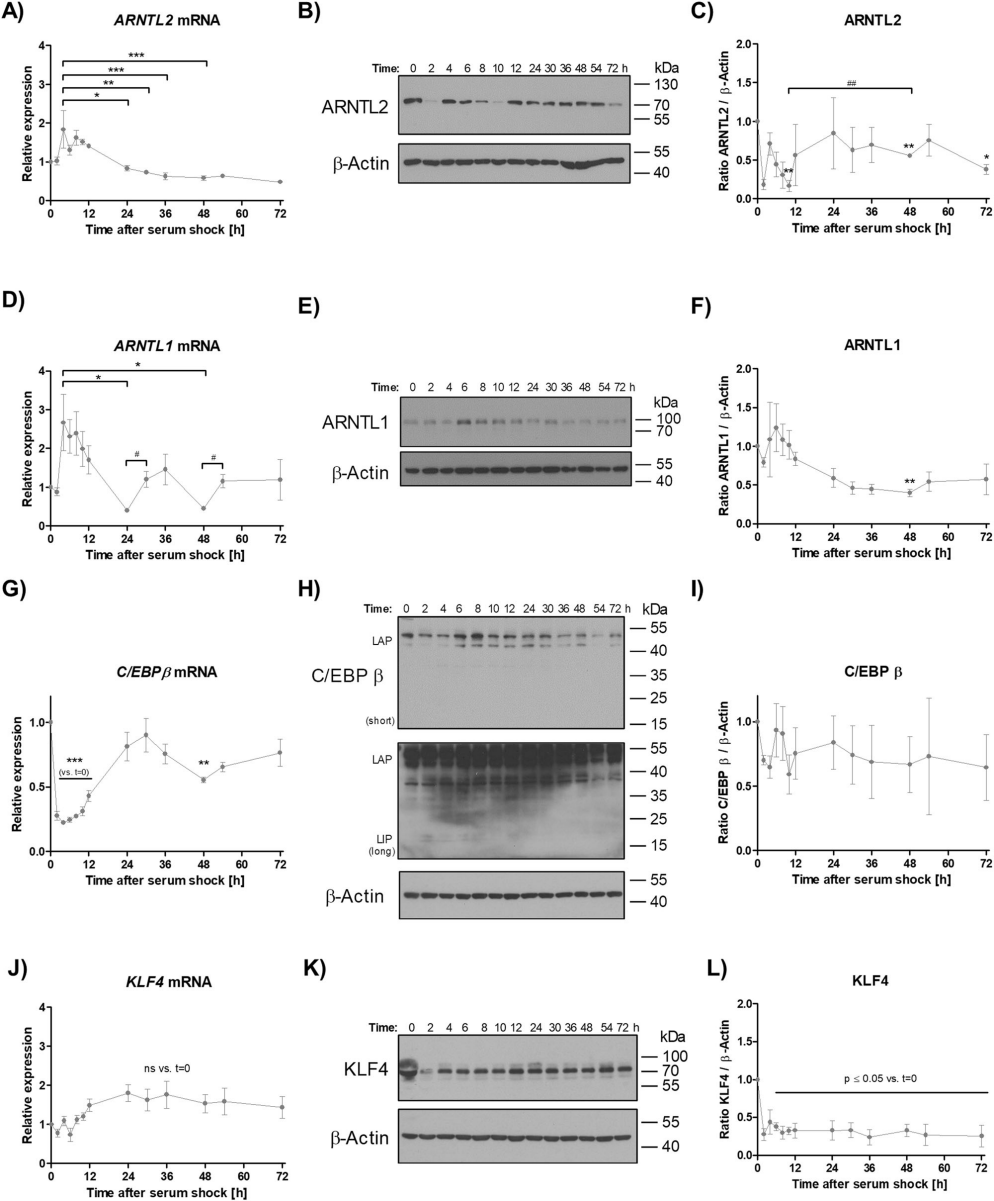
Induction of rhythmic gene expression by serum-shock in ASCs. **A** *ARNTL2* mRNA expression. Values are presented as mean ± SEM from i = 3 different donors. Statistical analysis was done using One-way ANOVA and Bonferroni´s multiple comparison test. **B** ARNTL2 Western blot analysis. A representative result of *n* = 3 different donors is shown. β-Actin served as loading control. **C** Densitometric analysis of ARNTL2 protein level. Values are presented as mean ± SEM from *n* = 3 different donors. Statistical comparison was achieved using the two-tailed paired *t* test (vs. *t* = 0; *) and the two-tailed unpaired *t* test (#). **D** *ARNTL1* mRNA expression. Values are presented as mean ± SEM from *n* = 3 different donors. Statistical analysis was done using One-way ANOVA and Bonferroni´s multiple comparison test (*) in addition to the two-tailed unpaired *t* test (#). **E** ARNTL1 Western blot analysis. A representative result of *n* = 3 different donors is shown. β-Actin served as loading control. **F** Densitometric analysis of ARNTL1 protein level. Values are presented as mean ± SEM from *n* = 3 different donors. Statistical comparison was done using One-way ANOVA and Bonferroni´s multiple comparison test. **G** *C/EBPB* mRNA expression. Values are presented as mean ± SEM from *n* = 3 different donors. Statistical analysis was achieved using One-way ANOVA and Bonferroni´s multiple comparison test. **H** C/EBP β Western blot. A representative result of *n* = 3 different donors is shown. Short: short exposure; long: long exposure (~1 h). β-Actin served as loading control. **I** Densitometric analysis of C/EBP β protein level. Values are presented as mean ± SEM from *n* = 3 different donors. **J** Response of KLF4 *KLF4* mRNA expression after circadian synchronization by serum-shock. Values are presented as mean ± SEM from *n* = 3 different donors. Statistical comparison was done with One-way ANOVA with Bonferroni´s multiple comparison test. **K** Representative KLF4 Western blot of *n* = 3 individual donors after serum-shock. β-Actin served as loading control. **L** Densitometric analysis of KLF4 protein level. Values are presented as mean ± SEM from *n* = 3 different donors. Statistical comparison was done with One-way ANOVA and Bonferroni´s multiple comparison test.

To investigate whether the expression of *ARNTL2* follows a circadian profile in ASCs we used a serum-shock approach, which is generally employed to induce rhythmic expression of appropriate genes [23, 37]. Confluent serum-starved ASCs were treated with 30 % FCS for 2 hours and subsequently cultured in serum-free medium up to 72 h. *ARNTL2* mRNA expression was induced and peaked 4 hours after serum-shock followed by a continuous decline (Fig. 2A), while ARNTL2 protein levels showed a clear oscillatory pattern in all donors tested (Fig. 2B, C). For comparison, *ARNTL1* mRNA expression exerted a rhythmic pattern with a peak induction 4 h after circadian synchronization followed by two cycles of alternating decline and re-induction (Fig. 2D). On protein level, ARNTL1 was highly abundant 6 h after serum-shock and declined thereafter (Fig. 2E, F). To calculate the circadian characteristics (i.e., mesor, amplitude, acrophase) of *ARNTL1* and *ARNTL2* Cosinor regression analysis was performed. This approach is applicable to investigate circadian rhythms in non-equidistant time series [38]. Overall, ARNTL1 mRNA expression was well reflected by a Cosinor function (*P*-value 0.054) (Supplementary Fig. S1) as also shown by other studies [39–41]. In contrast, the ARNTL2 mRNA regression analysis was less accurate (*P*-value 0.144) (Supplementary Fig. S1). In summary, these data show a different regulation of ARNTL1 and ARNTL2 in response to circadian cues.

Next, we asked whether the early adipogenic transcription factor C/EBPβ [42, 43] is affected by circadian synchronization in ASCs as demonstrated in other models [44]. Intriguingly, *C/EBPβ* mRNA showed a clear rhythmic expression pattern in response to serum-shock treatment (Fig. 2G) whereas this effect was not found on protein level (Fig. 2H, I). To better understand the underlying mechanism, we analyzed whether KLF4, a ASC-specific transcription factor known to drive *C/EBPβ* expression at the onset of adipogenic differentiation, is also oscillating after serum-shock [42]. As presented in Fig. 2J–L, neither RT-qPCR analysis nor Western blotting provided compelling evidence for rhythmic *KLF4* expression after circadian synchronization. We conclude, *C/EBPβ* gene expression is clock-controlled in ASCs but its protein level is not affected by serum-shock.

### ARNTL2 protein accumulates rapidly in ASCs stimulated with adipogenic differentiation medium

**Fig. 3:**
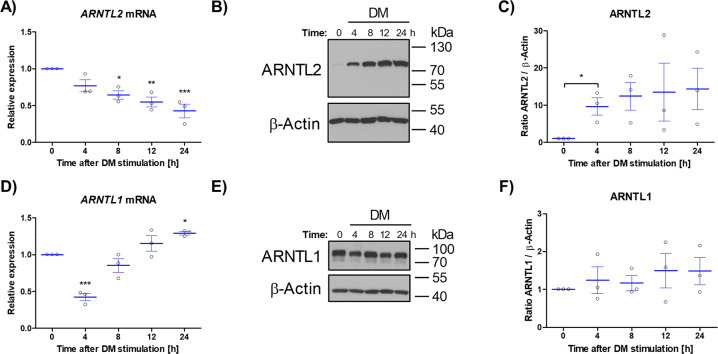
Impact of short-term stimulation of ASCs with adipogenic differentiation medium on ARNTL1 and ARNTL2 expression. **A** *ARNTL2* mRNA expression. Values are given as mean ± SEM of *n* = 3 individual donors. Statisitcal analysis between groups was achieved using One-way ANOVA with Dunnett´s multiple comparison test. **B** A representative ARNTL2 Western blot of *n* = 3 different donors is shown. β-Actin served as loading control. **C** Densitometric quantification of ARNTL2 Western blots of *n* = 3 individual donors. Values are shown as mean ± SEM. Statistical comparison was done with the paired *t* test. **D** *ARNTL1* mRNA expression. Values are given as mean ± SEM of *n* = 3 individual donors. Statisitcal analysis between groups was achieved using One-way ANOVA with Dunnett´s multiple comparison test. **E** A representative ARNTL1 Western blot of *n* = 3 different donors is shown. β-Actin served as loading control. **F** Densitometric quantification of ARNTL1 Western blots of *n* = 3 individual donors. Values are shown as mean ± SEM. Note: scaling of *y*-axis is different in (**C**) and (**F**). DM differentiation medium.

The different regulation of ARNTL1 and ARNTL2 by serum-shock (Fig. 2) prompted us to investigate the response of both transcription factors to adipogenic signals. Therefore, adipogenesis was stimulated with differentiation medium (DM) and *ARNTL2* expression monitored 4, 8, 12 and 24 h afterwards. The *ARNTL2* mRNA level declined continuously during the time-course investigated (Fig. 3A) whereas ARNTL2 protein abundance strongly increased within 4 h after stimulation and remained high (Fig. 3B, C). In contrast, *ARNTL1* mRNA expression was reduced 4 h post-treatment and increased thereafter (Fig. 3D) while the ARNTL1 protein level was not significantly altered among the donors tested (Fig. 3 E, F). In summary, these data indicate an unequal response of ARNTL1 and ARNTL2 to adipogenic stimuli which is consistent with our previous observation (Fig. 2).

### An ARNTL2-dependent feedback mechanism renders ASCs refractory to mTOR and MAPK signaling

**Fig. 4:**
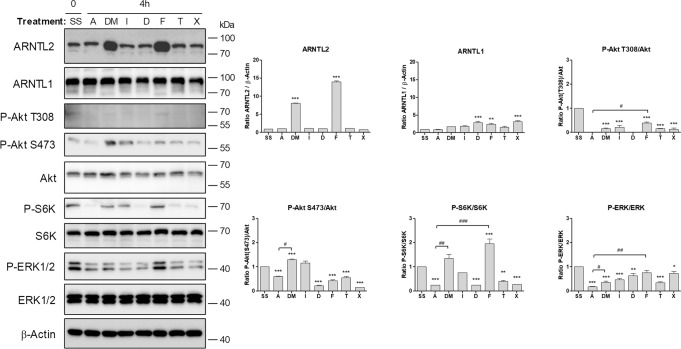
Induction of ARNTL2 by differentiation medium (DM) and single compounds. Confluent ASCs were serum-starved (SS) followed by treatment with serum-free ASC1 medium (A), differentiation medium (DM), insulin (I), dexamethasone (D), FCS (F), transferrin (T) and IBMX (X) for 4 h. Left panel: a representative Western blot of *n* = 3 independent experiments (i.e., donors) is shown. PI3K/Akt/mTOR and MAPK signaling was analyzed to confirm activity of each compound used. β-Actin served as loading control. Right panel: densitometric analysis of the Western blot shown on the left. Values are presented as mean ± SEM of three measurements. Statistical comparison was done using One-way ANOVA and Dunnett´s Multiple Comparison Test (vs. SS; *) and the two-tailed paired/unpaired *t* test (#).

To further elucidate the underlying mechanism of ARNTL2 upregulation in response to adipogenic cues, we evaluated the effects of individual DM components on the expression of ARNTL1 and ARNTL2. These experiments revealed that the addition of FCS (2.5% v/v) profoundly increased the protein level of ARNTL2 but not ARNTL1 after 4 h (Fig. 4). Moreover, we recognized that as expected PI3K/Akt, MAPK and mTOR signaling was activated under the same condition, as shown by increased phosphorylation of Akt Thr308, Akt Ser473, S6K1 and MAPK compared to corresponding ASC1 treated cells (Fig. 4).

**Fig. 5:**
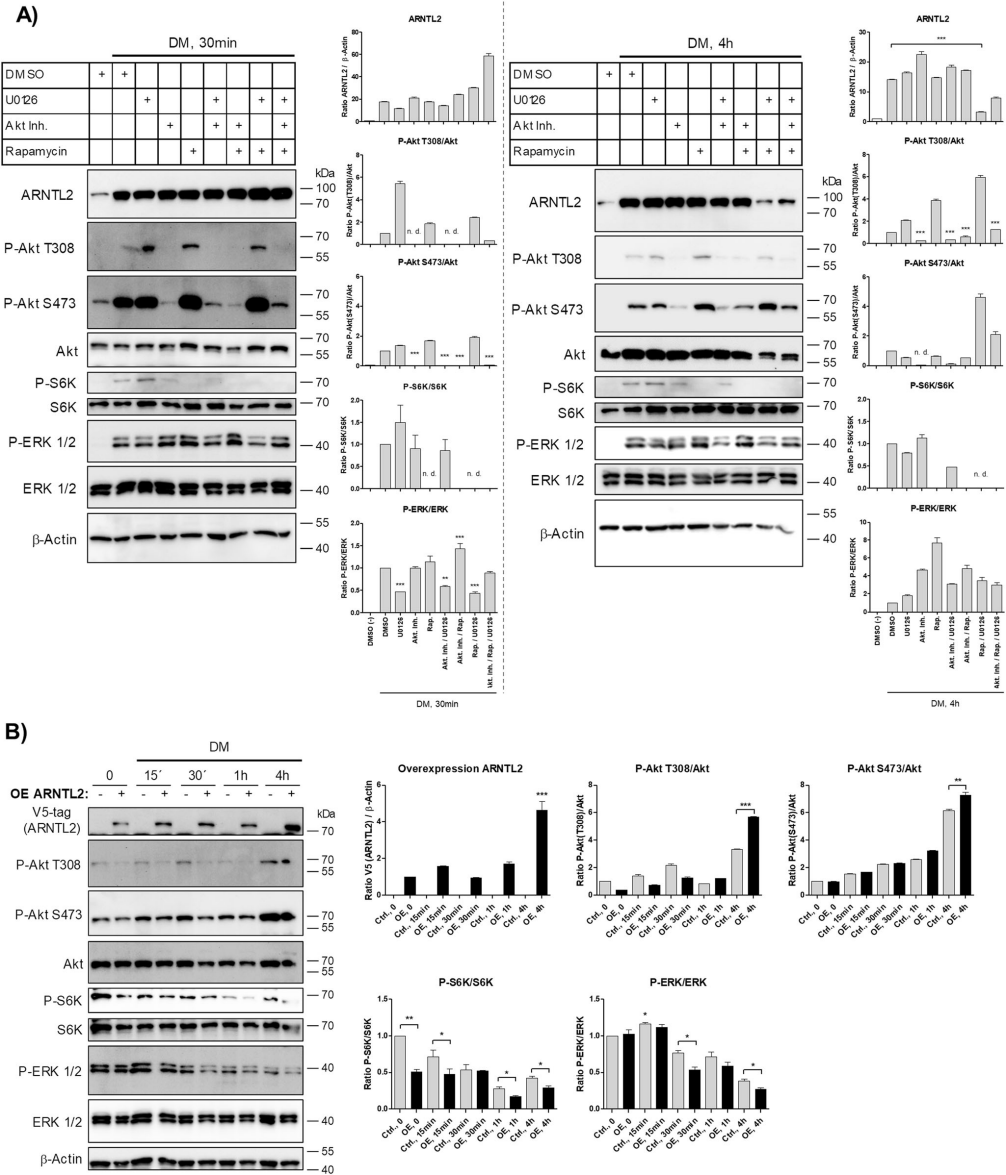
ARNTL2-dependent feedback mechanism. **A** Pharmacological inhibition of signaling pathways. Serum-starved ASCs were pre-treated with the indicated compounds or vehicle (DMSO) for 30 min followed by DM stimulation for 30 min (left panel) and 4 h (right panel), respectively. A representative Western blot and densitometry of *n* = 3 independent experiments (i.e., donors) is shown. β-Actin served as loading control. Values are presented as mean ± SEM of three measurements. Statistical comparison was done using One-way ANOVA and Dunnett´s Multiple Comparison test (vs. stimulated cells pre-treated with DMSO) and the unpaired two-tailed *t* test (between groups as indicated). **B** Stimulation of control (Ctrl.; −) and ARNTL2 overexpressing (OE; +) ASCs with DM for indicated time points. Left panel: A representative Western blot of *n* = 3 independent experiments (i.e., donors) is shown. β-Actin served as loading control. Right panel: Densitometry corresponding to the Western blot shown on the left. Values are presented as mean ± SEM of three measurements. Statistical comparison was done with One-way ANOVA with Dunnett´s Multiple Comparison test (vs. *t* = 0) or the two-tailed paired and unpaired *t* test between groups as indicated.

To analyze whether there is a causal relationship between these signaling circuits and DM-mediated ARNTL2 induction, we conducted pharmacological inhibition of these pathways. As presented in Fig. 5A (left panel), inhibition of neither PI3K/Akt/mTOR nor MAPK signaling alone or in combination prevented DM-dependent ARNTL2 upregulation after 30 min. Intriguingly, ARNTL2 protein was depleted in ASCs co-treated with U0126 and Rapamycin after 4 h (Fig. 5A, right panel). These results reveal that ARNTL2 stability is controlled by both the PI3K/Akt/mTOR and MAPK pathway and simultaneous inhibition of the two circuits is necessary to accelerate ARNTL2 degradation. We have previously shown that WL interventions induce the PI3K-inhibitor DIRAS3 and the Ras-inhibitor Sprouty1 as WL target genes, leading to down-regulation of both PI3K-mTOR and MAPK signaling in ASCs [13, 14, 34, 35]. To test whether ARNTL2 influences these pathways vice versa, ARNTL2 was ectopically overexpressed (OE) in ASCs followed by stimulation with DM for various time points. ARNTL2 OE ASCs showed an impaired mTOR and MAPK activity as evidenced by reduced P-S6K/S6K and P-ERK/ERK ratios compared to control cells (Fig. 5B). In conclusion, these results suggest the existence of a feedback mechanism in which ARNTL2 stability is maintained in a PI3K/Akt/mTOR and MAPK dependent manner whereas ARNTL2 acts as an inhibitor on these pathways.

### *ARNTL2* overexpression inhibits adipogenesis by inducing degradation of ARNTL1, down-regulation of the MAPK-*C/EBPβ* axis and attenuation of *KLF15* gene expression

**Fig. 6:**
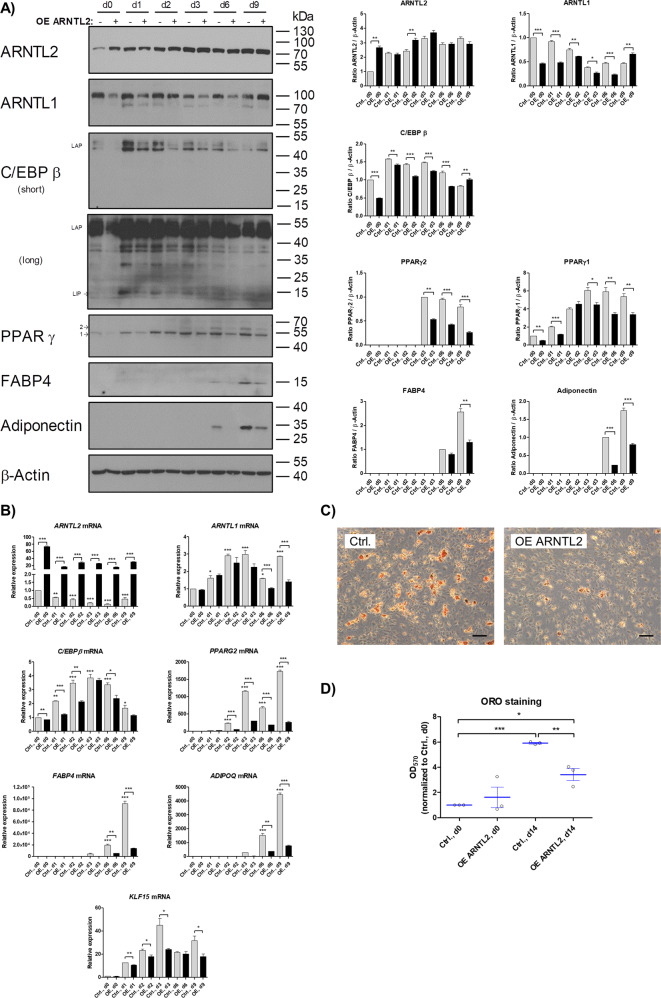
Effects of *ARNTL2* overexpression on adipogenesis. **A** Western blot analysis of control ASCs (Ctrl., −), harboring the empty vector, and *ARNTL2* overexpressing (OE, + ) ASCs subjected to adipogenic differentiation. Left panel: representative Western blots of ASCs from *n* = 3 donors are shown. β-Actin served as loading control. Right panel: Densitometric quantitation of the Western blots is shown. Values are presented as mean ± SEM of three measurements. Statistical comparison was achieved using the Paired *t* test (vs. normalized group) and the Unpaired *t* test. **B** RT-qPCR analysis of adipogenic marker genes. Representative result of *n* = 3 donors. Values are presented as mean ± SEM of three technical replicates. Statistical comparison within one group was done with One-way ANOVA and Dunnett´s multiple comparison test. Statistical comparison between groups was achieved using the paired *t* test (vs. normalized control group) and the unpaired *t* test. **C** Oil Red O staining of control ASCs (Ctrl.) harboring the empty vector and *ARNTL2* overexpressing (OE) ASCs on d14 of adipogenic differentiation. A representative result of *n* = 3 different donors is shown. Microphotographs were taken at ×50 magnification. Scale bar: 200 µm **D** Quantification of Oil Red O staining. Values are presented as mean ± SEM of *n* = 3 different donors. Statistical comparison between groups was achieved using the paired *t* test (vs. normalized control group) and the unpaired *t* test.

To analyze whether an elevated basal level of ARNTL2 affects adipogenesis, the circadian transcription factor was overexpressed in ASCs and adipogenic differentiation induced by DM (Fig. 6). Overexpression of ARNTL2 led to reduced adipogenic differentiation, as shown by a considerable reduced expression of the mRNA of the early adipogenic regulator *C/EBPβ*, the adipogenic key-regulator *PPARγ2* and the adipocyte marker genes *FABP4* and *ADIPOQ* (Fig. 6B). ARNTL2 overexpressing cells show also a significant decrease of the C/EBPβ, PPARγ2, FABP4 and Adiponectin protein level relative to control cells (Fig. 6A, panels 3–7). Moreover, the accumulation of intracellular lipids was significantly reduced in ARNTL2 overexpressing ASCs underscoring the inhibitory effect of this transcription factor on adipogenesis (Fig. 6C, D).

**Fig. 7:**
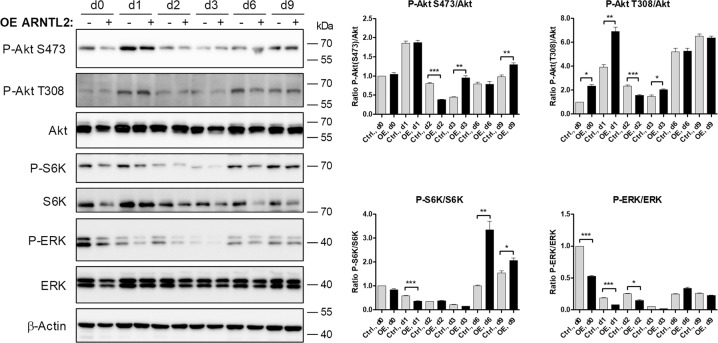
PI3K/Akt/mTOR and MAPK signaling during adipogenesis in *ARNTL2* OE ASCs. Corresponding result to Fig. 6A. See legend of Fig. 6 for details.

To further elucidate the underlying mechanism, we analyzed the effects on PI3K/Akt/mTOR and MAPK signaling. Consistent with our previous experiments (Fig. 5), we found that ARNTL2 OE ASCs showed a strong decrease in MAPK signaling at the onset of terminal adipogenesis (Fig. 7). These results underscore that an elevated ARNTL2 protein level leads to profound down-regulation of MAPK signaling in ASCs in response to adipogenic signals, correlating with decreased level of the early adipogenic transcription factor *C/EBPβ* (Fig. 6). This finding provides one mechanism how ARNTL2 impairs adipogenesis.

Moreover, the ARNTL2 OE ASCs showed an activation of AktThr308 signaling at certain time points during adipogenesis and later on in premature adipocytes AktSer473 phosphorylation was increased and mTOR signaling activated (Fig. 7). This finding indicates that the suppressive effect of ARNTL2 (Fig. 6) is abolished several days after induction of adipogenesis.

ARNTL1 is a positive regulator of adipogenesis [17, 45]. Consistently, the ARNTL1 protein level were strongly decreased in *ARNTL2* OE ASCs compared to control cells until day 6 after induction of differentiation while *ARNTL1* mRNA expression was not significantly different between both cell populations during differentiation up to day 3 (Fig. 6A, B). This provides another mechanism contributing to ARNTL2-mediated inhibition of adipogenesis and indicates that a post-transcriptional and/or post-translational mechanism leads to the reduced ARNTL1 protein levels in *ARNTL2* OE ASCs.

To analyze whether proteolysis plays a role, we measured the half-life of ARNTL1 protein in ARNTL2 OE ASCs on day 1 of adipogenesis. As shown in Supplementary Fig. S2A, ARNTL2 OE reduced the ARNTL1 basal level as expected and decreased its stability as evidenced by a shorter half-life. Employment of MG132 rescued the ARNTL1 protein level in both control and ARNTL2 OE cells to a similar extent, indicating that ARNTL2-induced ARNTL1 degradation is mediated by the ubiquitin-proteasome pathway (Supplementary Fig. S2A).

Based on the finding of ARNTL2-mediated ARNTL1 deprivation, we asked whether ARNTL2 OE might also inhibit *PER3* or *KLF15* expression, which were shown to provide a link between the circadian clock and adipogenesis [46]. *Aggarwal et al* [46]. demonstrated that *KFL15* is directly regulated in an ARNTL1 and PER3 dependent manner [46]. Indeed, *KLF15* mRNA was significantly less abundant in ARNTL2 OE ASCs compared to control cells whereas *PER3* was not detectable in all samples (Fig. 6B).

In conclusion, these data demonstrate an antagonistic relationship between ARNTL1 and ARNTL2 during adipogenic differentiation. The finding that ARNTL2 accumulates in ASCs in response to adipogenic cues (Figs. 3–6) together with its anti-adipogenic mode of action (Fig. 6), reveals its role as an inducible inhibitor of this process.

### ARNTL2 protein is highly abundant in sWAT from WLDs

**Fig. 8:**
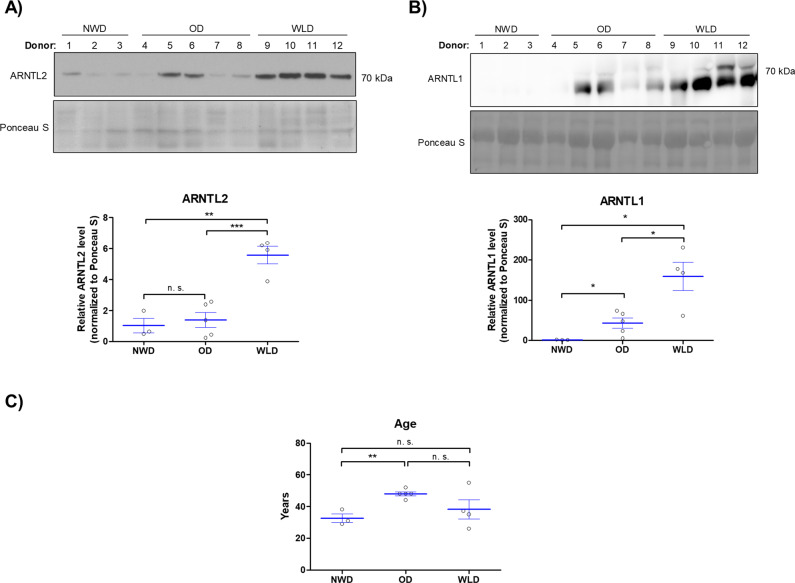
Western blot analysis of sWAT lysates of NWDs, ODs and WLDs. **A** ARNTL2 protein levels were detected by Western blot analysis, quantified and normalized to Ponceau S staining. Statistical comparison was done using the two-tailed unpaired *t* test. **B** ARNTL1 protein levels were detected by Western blot analysis, quantified and normalized to Ponceau S staining. Statistical comparison was done using the two-tailed unpaired *t* test. **C** Age of donors corresponding the result presented in (**A**) and (**B**). Statistical comparison was done with the two-tailed unpaired *t* test.

Initially, we identified *ARNTL2* as a WL target gene in human ASCs that is strongly down-regulated at the mRNA level in WLDs (Fig. 1). However, we recognized an increase in the ARNTL2 protein levels in the course of adipogenesis in ASCs derived from WLDs resulting in very high ARNTL2 protein level in in vitro differentiated adipocytes (Fig. 6). Moreover, we observed a high stability of the ARNTL2 protein in differentiating and proliferating ASCs (Supplementary Fig. S2). Therefore, we analyzed whether this transcription factor might be elevated in adipose tissue samples of WLDs. In fact, Western blot analysis of whole sWAT samples derived from NWDs, ODs and WLDs revealed significantly higher ARNTL2 protein levels in WLDs compared to the other two groups (Fig. 8A). In contrast, ARNTL1 was significantly increased in ODs and WLDs compared to NWDs (Fig. 8B). The age of the donors had no influence on the ARNTL2 protein level (Fig. 8C). These findings are in agreement with our results shown above in adipocytes (Fig. 6) and underscore that WL interventions induce an elevation of ARNTL2 protein level in sWAT.

## Discussion

Disruption of physiological circadian rhythms predisposes to obesity and premature aging [3, 22, 47–49]. Thereby changes in human WAT, in which approximately 25% of the transcriptome shows a diurnal variation [50], play an important role. In the present study we elucidated the role of the circadian transcription factor ARNTL2 in adipogenesis.

Initially, to analyze circadian gene expression in ASCs we applied a well-established serum-shock approach [23, 37]. ARNTL2 protein showed a clear oscillatory pattern in contrast to the corresponding mRNA, suggesting a post-translational regulation of ARNTL2 (Fig. 2). *ARNTL1* mRNA exerted a pronounced circadian expression pattern after serum-shock (Fig. 2 and Supplementary Fig. S1) as also demonstrated by others [23]. In contrast to ARNTL2, ARNTL1 showed no obvious oscillation on protein level. These findings reveal a different response of both transcription factors to circadian synchronization thus arguing against a co-regulation of ARNTL1 and ARNTL2 in ASCs as proposed in other models [20]. In agreement with this observation, our results reveal opposite effects of ARNTL1 and ARNTL2 proteins in response to adipogenic signals. Short-term stimulation of ASCs with differentiation medium or only with FCS increased ARNTL2 protein (Figs. 3, 4) whereas ARNTL1 showed no clear effect among the donors examined (Fig. 3).

In the course of adipogenesis, ARNTL2 protein was rapidly elevated, highly stable and maintained at constant levels (Figs. 3–6). Consistently, our inhibition experiments revealed that ARNTL2 is sustained cooperatively in a mTOR and MAPK-dependent manner (Fig. 5). In agreement with our results, a previous study demonstrated that mTOR inhibition activates protein degradation by the ubiquitin proteasome system and autophagy, thereby accelerating the destruction of especially long-lived proteins [51]. Another study showed that suppression of the MAPK pathway also promotes protein degradation under certain conditions [52]. Interestingly, in our study, suppression of either the mTOR or the MAPK cascade alone had no effect on ARNTL2 protein stability (Fig. 5). This indicates that both pathways converge at ARNTL2. Indeed, a complex crosstalk and compensation mechanism among mTOR and MAPK signaling exists [53]. On the other hand, ARNTL2 OE ASCs exhibited an impaired mTOR and MAPK activation compared to control cells (Fig. 5B). This finding reveals ARNTL2 as a negative regulator of both circuits whereas the underlying mechanism needs to be analyzed in future studies. In summary, the maintenance of ARNTL2 by mTOR and MAPK activity together with its suppressive function on these pathways, provides an explanation of the constant levels of this transcription factor during adipogenesis.

Adipogenesis is controlled by the circadian clock as demonstrated by previous studies [17, 46] and depletion of ARNTL1 is known to inhibit this process [17]. Consistently, ARNTL2 OE induced ARNTL1 proteolysis leading to impaired adipogenic differentiation (Fig. 6; Supplementary Fig. S2). Moreover, *Aggarwal et al* [46]. identified the pro-adipogenic transcription factor KLF15 to be an ARNTL1 target gene [46]. In agreement, *KLF15* mRNA expression was significantly decreased in differentiating ARNTL2 OE ASCs (Fig. 6). *Matoba et al* [54]. reported that adipose-specific deletion of KLF15 decreased adiposity and conferred a protective effect against diet-induced obesity [54]. Therefore, our data indicates that ARNTL2 is a negative regulator of adipogenesis (i.e., hyperplasia) and probably also of adipocyte hypertrophy as suggested by the high abundance of ARNTL2 solely in sWAT samples obtained from WL donors (Fig. 8). Noteworthy, ARNTL1 was elevated in OD and WLD sWAT samples compared to NWD controls (Fig. 8). Taken together, these findings imply that the ARNTL1/ARNTL2 system is an important regulator of WAT homeostasis.

CR is a weight-loss intervention that mediates health- and life-prolonging effects [28, 55] and has been shown to facilitate stress adaptation in stem cells [36, 56, 57]. Previously our group identified several WL target genes such as *SPRY1*, a negative regulator of the MAPK cascade, and *DIRAS3*, an inhibitor of PI3K/Akt/mTOR signaling, and demonstrated their importance for proper ASC proliferation and differentiation [13, 14, 34, 35]. These studies support the concept that induction of *SPRY1* and *DIRAS3* in ASCs due to WL interventions confers a protective effect for fasting individuals by downregulation of MAPK and PI3K/Akt/mTOR signaling, thereby attenuating cellular senescence [13, 14, 34, 35]. In accordance with our former reports, ARNTL2 OE ASCs exhibited impaired mTOR and MAPK activity upon stimulation **(**Fig. 5**)**. Therefore, our results suggest that increased ARNTL2 protein levels in ASCs might protect them from aberrant proliferation and cellular senescence.

Whether the depletion of ARNTL2 promotes cellular aging remains an unsolved question. Employment of specific shRNAs abolished *ARNTL2* mRNA expression but failed to significantly reduce the corresponding protein level (data not shown). A CRISPR/Cas9-mediated gene knockout approach, as recently described by us [58], was ineffective despite the use of several target sequences (data not shown). The failure of both techniques to profoundly reduce the ARNTL2 protein level in ASCs is most likely due to the very high stability of this transcription factor (Supplementary Fig. S2).

Overall, we recognized an inverse correlation between *ARNTL2* mRNA and protein levels upon stimulation (Figs. 2, 3, 6). A high abundance of ARNTL2 protein was associated with downregulation of the corresponding mRNA which is a common pattern of auto-regulatory circuits [59, 60]. Intriguingly, similar effects have been described for ARNT [61–66]. Whether *ARNTL2* expression is controlled by an auto-regulatory mechanism needs to be investigated in future studies.

Physiologically, food consumption is an important time cue in humans [8] and capable to influence circadian clocks of peripheral tissues such as WAT [6, 67]. Therefore, time-related dietary interventions are considered as a strategy to treat obesity [4]. Our results support the hypothesis, that at certain time-points during the day, i.e., when ARNTL2 protein reached a high level due to circadian regulation, ASCs are refractory to adipogenic signals. Indeed, the internal circadian time in humans can be accurately measured as demonstrated by *Wittenbrink et al* [68]. Further studies are required to investigate the linkage between food intake, ARNTL2 abundance in ASCs and adipogenesis, which might lead to a new strategy to combat obesity.

In conclusion, our results reveal major differences of the clock transcription factors ARNTL1 and ARNTL2 in human ASCs regarding their response to circadian/adipogenic signals, stability and function. We demonstrate an antagonistic relationship between ARNTL1 and ARNTL2 during adipogenesis and identify ARNTL2 to be an inducible inhibitor of this process.

## Materials and methods

### Donor characteristics

Subcutaneous white adipose tissue (sWAT) samples were obtained from patients undergoing routine elective plastic abdominal surgery at the Department of Plastic, Reconstructive and Aesthetic Surgery, Medical University of Innsbruck, Austria. All patients gave their informed written consent. The study protocol was approved by the Ethics Committee of the Medical University of Innsbruck (Austria) according to the Declaration of Helsinki. For the present study, sWAT samples taken from the lower abdomen (i.e., from the layer between fascia of scarpa and rectus fascia) of *n* = 32 healthy donors of Caucasian origin were used (Supplementary Table 1).

### Isolation, cultivation, and differentiation of human ASCs

ASCs were isolated and cultured as described in *Hatzmann et al., 2021* [69]. Adipogenic differentiation was carried out as explained in *Ejaz et al. 2017* [14].

### sWAT whole tissue lysates

To gain protein samples, 0.2-1 g of sWAT was mixed with 500 µl lysis buffer (1% v/v NP40 in PBS supplemented with cOmplete™ EDTA-free Inhibitor Cocktail (Roche)), homogenized with a Dounce homogenisator on ice and sonicated. The lysates were centrifuged (13.000 rpm, 10 min, 4 °C) and the aqueous phase was transferred into a new tube followed by protein determination. Finally, samples were subjected to Western blotting.

### *ARNTL2* overexpression (OE)

For ectopic overexpression of *ARNTL2*, an appropriate pENTR223 plasmid containing the *ARNTL2* cDNA was purchased from the DNASU Plasmid Repository (HsCD00514112) and cloned into the pLenti6/V5-DEST vector by recombination using the Gateway® System (Invitrogen) in accordance with the manufacturer´s protocol. The final plasmid pLenti6-ARNTL2-V5 was confirmed by restriction digest and sequencing. For control, the empty vector was employed [70]. All plasmids were amplified in *E. coli Stbl3* bacteria. Endotoxin-free plasmid preparations for transfection were gained with the EndoFree® Plasmid MaxiKit (Qiagen) or the GeneJET Endo-free Plasmid Maxiprep Kit (Thermo Scientific) as described in the supplier´s guidelines.

### Lentiviral particles and infection of ASCs

Generation of lentiviral particles and infection of ASCs was done as described in Mandl et al. [13].

### Serum-shock approach

Rhythmic gene expression in ASCs was induced by serum-shock as previously demonstrated by *Wu et al*. [23],. For this purpose, ASCs were seeded in 6-well plates at a density of ~1 × 10^4^ cells/cm^2^ and cultured until confluence. ASCs were serum-starved for two days followed by treatment with 30% v/v FCS for 2 h (defined as *t* = 0). Subsequently, the supernatant was replaced by serum-free. Cells were harvested at certain time points after serum-shock (i.e., 0, 2, 4, 6, 8, 10, 12, 24, 30, 36, 48, 54, and 72 h) to gain RNA and protein samples, respectively. Circadian parameters of core clock genes were determined by Cosinor regression analysis which is a suitable method for non-equidistant data sets [38] and known to describe the ARNTL1 mRNA rhythm [39–41]. Cosinor calculation was done using time points 2-24 h by employing a web-based tool (https://cosinor.online/app/cosinor.php) [71].

### Pharmacological inhibition of signaling pathways

ASCs were seeded at a density of ~1 × 10^4^ cells/cm^2^ in 6-well plates and grown until confluence followed by serum-starvation for two days. Subsequently, ASCs were pre-treated with the specific inhibitors (Akt Inhibitor VIII (Calbiochem; 10 µM); U0126 (SigmaAldrich; 50 µM); Rapamycin (Merck; 0.5 µM)) for 30 min or DMSO (1% v/v) diluted in serum-free ASC1 medium. Afterwards, ASCs were stimulated with 2x differentiation medium (DM) and harvested after 30 min and 4 h, respectively.

### Western blot analysis

Western blotting was performed as described [13]. Primary antibodies are listed in Supplementary Table 2. Polyvinylidene fluoride (PVDF) membranes were finally exposed to an X-ray film (Super RX-N, FUJI) or analyzed using the ChemiDoc™ Imaging System (BioRad). Signals were quantified with ImageJ (version 1.47; National Institutes of Health, USA) or Image Lab™ software (version 6.0.1; BioRad). The specificity of the anti ARNTL2 antibody was verified by 2-dimensional (2D) gel-electrophoresis as described below and shown in Supplementary Fig. S3. Uncropped Western blot results are provided as Supplemental Material.

### 2-dimensional (2D) gel-electrophoresis

ARNTL2 overexpressing and Blasticidin-selected ASCs were seeded in 2 × 15 cm cell culture dishes and grown until confluence. Cells were washed twice with ice-cold PBS, harvested by scraping in PBS and transferred into 1.5 ml tubes. Subsequently, cells were centrifuged (30 sec; 13.000 rpm) and the supernatant removed. Pellets were suspended in 200 µl ice-cold PBS and pooled. To prevent degradation of proteins, Halt™ Protease Inhibitor Cocktail 100x (Thermo Scientific) was added. Samples were sonicated and the protein concentration was determined. Next, ~200 µg of protein were loaded onto Immobiline® DryStrips (pH 3-10; 7 cm; NL; Sigma Aldrich) and isoelectric focusing was done in accordance with the manufacturer´s guidelines followed by Sodium Dodecyl Sulfate (SDS) – Polyacrylamide gel electrophoresis (PAGE) and Western blotting.

### Measurement of protein half-life

Protein turn-over was measured by Cycloheximide (CHX; Biomol) chase assays. Proliferating and differentiating ASCs were treated with 100 µg/ml and 300 µg/ml CHX, respectively, for various time points and analyzed by Western blotting. Protein half-life was calculated as described [72]. The proteasome inhibitors MG132 (Sigma Aldrich) and LLnL (Sigmal Aldrich) were employed for rescue experiments [73, 74].

### Microarrays

Microarrays were performed in Ejaz et al. [34].

### Gene expression analysis

RNA isolation and cDNA synthesis was done as previously described [75]. Gene expression was measured using the QuantStudio 7 Real-Time PCR system (AppliedBiosystems) and SYBR green chemistry (AceQ qPCR SYBR Green, Vazyme Biotech). Primer sequences are provided in Supplementary Table 3. Changes in gene expression were calculated using the Comparative relative quantification (ΔΔC_T_) method with β-Actin (*ACTB*) as endogenous reference.

### Quantification of intracellular lipids

Quantification of lipid accumulation was carried out as described [13].

### Online databases

Detailed information regarding ARNTL1 and ARNTL2 were retrieved from the BLAST (https://blast.ncbi.nlm.nih.gov/Blast.cgi), ExPASy (https://www.expasy.org/) and UniProt (https://www.uniprot.org/) databases.

### Statistical analysis

Statistics was done with GraphPad Prism 5 software (GraphPad Software Inc., La Jolla, CA, USA). Each experiment was performed with a minimum of *n* = *3* biological replicates (i.e., donors). All measurements were done in triplicates. Values are presented as mean ± SEM. Statistical comparison was achieved using the paired or unpaired two-tailed *t* test or ANOVA depending on the type of data set and as mentioned in the corresponding figure legend. *p* values ≤ 0.05 were considered to be significant as indicated: *p < 0.05; **p < 0.01; ***p < 0.001.

## Supplementary information

Supplementary Figure Legend S1

Supplementary Figure Legend S2

Supplementary Figure Legend S3

Supplementary Table 1

Supplementary Table 2

Supplementary Table 3

Supplementary Figure S1

Supplementary Figure S2

Supplementary Figure S3

Original Data File

## Data Availability

All data generated or analyzed during this study are included in this article and its supplementary files. Uncropped Western blot results are provided as Supplemental Material.
