# Supplementary material for: The circadian transcription factor ARNTL2 is regulated by weight-loss interventions in human white adipose tissue and inhibits adipogenesis
Source: Cell Death Discov. 2022 Nov 3;8:443. doi: 10.1038/s41420-022-01239-3 (PMC9633602; doi:10.1038/s41420-022-01239-3)
Supplement: Supplementary file 1 — Supplementary Figure Legend S1 [file 41420_2022_1239_MOESM1_ESM.docx]

**Supplementary Figure S1:** Cosinor analysis of ARNTL1 and ARNTL2 mRNA expression to determine circadian characteristics. ARNTL1 and ARNTL2 mRNA expression data presented in Figure 2 and within the time frame 2-24h were subjected to Cosinor regression analysis using a web-based application (https://cosinor.online/app/cosinor.php) ([1](#_ENREF_1)). The curve-fit results for each individual donor are shown (grey lines). Mean mRNA expression was calculated among donors prior Cosinor analysis (ARNTL1: blue line; ARNTL2: red line). Ampl.: Amplitude; Acro.: Acrophase;

**Supplementary References**

1. Molcan L. Time distributed data analysis by Cosinor.Online application. bioRxiv. 2019:805960.
