# Supplementary material for: The circadian transcription factor ARNTL2 is regulated by weight-loss interventions in human white adipose tissue and inhibits adipogenesis
Source: Cell Death Discov. 2022 Nov 3;8:443. doi: 10.1038/s41420-022-01239-3 (PMC9633602; doi:10.1038/s41420-022-01239-3)
Supplement: Supplementary file 2 — Supplementary Figure Legend S2 [file 41420_2022_1239_MOESM2_ESM.docx]

**Supplementary Figure S2:** Analysis of ARNTL1 and ARNTL2 turn-over. A) Differentiating ASCs: Protein degradation was measured by treatment of confluent ASCs on d1 of adipogenesis with Cycloheximide (CHX) for indicated time points followed by Western blotting. Left panel: A representative result of n=3 different experiments (i.e., donors) is shown. β-Actin served as loading control. Molecular masses are given in kDa. Right panel: Densitometric analysis of the blot shown on the left. Values are given as mean +/- SEM of three measurements and were normalized to t=0.5h to ensure proper pre-incubation of CHX. Statistical comparison was done with the two-tailed paired t test. Protein half-life was calculated as described in the Methods section. B) Proliferating ASCs: Left panel: ARNTL2 and ARNTL1 protein level following CHX treatment over time. Representative Western blots out of n=5 different donors are shown. β-Actin served as loading control. Molecular masses are given in kDa. Right panel: Densitometry. Values are presented as mean +/- SEM of n=5 donors and were normalized to t=0.5h to ensure proper pre-incubation of the compound. n. a.: not applicable;
