# Supplementary material for: The circadian transcription factor ARNTL2 is regulated by weight-loss interventions in human white adipose tissue and inhibits adipogenesis
Source: Cell Death Discov. 2022 Nov 3;8:443. doi: 10.1038/s41420-022-01239-3 (PMC9633602; doi:10.1038/s41420-022-01239-3)
Supplement: Supplementary file 3 — Supplementary Figure Legend S3 [file 41420_2022_1239_MOESM3_ESM.docx]

**Supplementary Figure S3:** The anti-ARNTL2 antibody (NBP2-32423, Novus Biologicals) shows no cross-reactivity with ARNTL1. The *ARNTL2* gene is evolved by duplication of *ARNTL1* ([1](#_ENREF_1)). Since the two human transcription factors share 49 % amino acid identity ([2](#_ENREF_2)) we aimed to re-confirm the specificity of the antibody used by excluding cross-reactivity with ARNTL1. A) Overexpression of V5-tagged ARNTL2 in ASCs. Western blot analysis using an antibody against the C-terminal V5-epitope tag to confirm the abundance of the ectopically overexpressed ARNTL2 protein. β-Actin was used as loading control. A representative result of n=3 independent experiments (i.e., donors) is shown. B) Western blot analysis using the selected anti-ARNTL2 antibody to detect the V5-tagged ARNTL2 protein overexpressed in ASCs. β-Actin was used as loading control. A representative result of n=3 independent experiments (i.e., donors) is shown. C) 2D gel-electrophoresis of V5-tagged ARNTL2 overexpressing ASCs. Protein samples were subjected to isoelectric focusing followed by SDS-PAGE and Western blotting. Left panel: probing the membrane with the anti-ARNTL2 antibody generated one dot (white arrow) at the predicted isoelectric point of ARNTL2 (pI ~7). Middle panel: re-probing the same membrane with an anti-ARNTL1 antibody (anti-BMAL1, #14020, Cell Signaling Technology) resulted in the appearance of another dot (grey arrow) at the expected isoelectric point of ARNTL1 (pI ~6.4). The white arrow indicates the remaining ARNTL2 signal thereby confirming the specificity of the anti-ARNTL2 antibody which shows no cross-reactivity with ARNTL1. Right panel: Coomassie stain of the Western blot membrane. A representative result of n=2 experiments is shown.

**Supplementary References**

1. Okano T, Sasaki M, Fukada Y. Cloning of mouse BMAL2 and its daily expression profile in the suprachiasmatic nucleus: a remarkable acceleration of Bmal2 sequence divergence after Bmal gene duplication. Neuroscience letters. 2001;300(2):111-4.

2. Ikeda M, Yu W, Hirai M, Ebisawa T, Honma S, Yoshimura K, et al. cDNA cloning of a novel bHLH-PAS transcription factor superfamily gene, BMAL2: its mRNA expression, subcellular distribution, and chromosomal localization. Biochemical and biophysical research communications. 2000;275(2):493-502.
