# Supplementary material for: The circadian transcription factor ARNTL2 is regulated by weight-loss interventions in human white adipose tissue and inhibits adipogenesis
Source: Cell Death Discov. 2022 Nov 3;8:443. doi: 10.1038/s41420-022-01239-3 (PMC9633602; doi:10.1038/s41420-022-01239-3)
Supplement: Supplementary file 4 — Supplementary Table 1 [file 41420_2022_1239_MOESM4_ESM.docx]

**Supplementary Table 1:** Characteristics of normal-weight donors (NWDs), obese donors (ODs) and weight-loss donors (WLDs). Human sWAT samples were obtained from the lower abdomen of donors undergoing routine abdominoplasty at the Department of Plastic, Reconstructive and Aesthetic Surgery at the Medical University of Innsbruck, Austria. BMI: Body mass index; f = female, m = male, n. a. = not available. ^#^) Used for Microarray analysis.

| Donor | Sex | Age [years] | BMI [kg/m^2^] | Treatment group |
| --- | --- | --- | --- | --- |
| 1 | m | 18 | 24,49 | WLD |
| 2 | f | 21 | 25,04 | WLD |
| 3^#^ | f | 23 | 19,83 | N |
| 4 | f | 24 | 20,31 | WLD |
| 5 | f | 26 | 22,47 | WLD |
| 6^#^ | f | 27 | 20,2 | WLD |
| 7 | f | 28 | 24,22 | WLD |
| 8 | f | 28 | 21,6 | WLD |
| 9 | f | 28 | 22,86 | WLD |
| 10^#^ | f | 29 | 23,71 | N |
| 11 | f | 29 | 22,99 | N |
| 12 | f | 31 | 25,18 | N |
| 13 | f | 33 | 24,09 | WLD |
| 14 | f | 34 | 25,95 | WLD |
| 15 | f | 34 | 25,95 | WLD |
| 16 | f | 35 | 22,23 | WLD |
| 17^#^ | f | 36 | 24,98 | WLD |
| 18 | f | 37 | 23,83 | WLD |
| 19 | f | 37 | 23,05 | WLD |
| 20^#^ | f | 38 | 24,61 | N |
| 21 | f | 38 | 24,45 | WLD |
| 22^#^ | f | 44 | 31 | O |
| 23^#^ | f | 45 | 22,27 | WLD |
| 24^#^ | f | 48 | 33,06 | O |
| 25^#^ | f | 48 | 31,99 | O |
| 26 | f | 48 | 34,18 | O |
| 27 | f | 50 | 27,99 | WLD |
| 28 | f | 52 | 38,57 | O |
| 29^#^ | f | 55 | 26,03 | WLD |
| 30 | f | 55 | 25,95 | WLD |
| 31 | f | 59 | 27,22 | WLD |
| 32 | f | 61 | 26,11 | WLD |
