# Supplementary material for: The circadian transcription factor ARNTL2 is regulated by weight-loss interventions in human white adipose tissue and inhibits adipogenesis
Source: Cell Death Discov. 2022 Nov 3;8:443. doi: 10.1038/s41420-022-01239-3 (PMC9633602; doi:10.1038/s41420-022-01239-3)
Supplement: Supplementary file 5 — Supplementary Table 2 [file 41420_2022_1239_MOESM5_ESM.docx]

**Supplementary Table 2:** Antibodies for Western blotting.

| Antibody | Supplier | Catalogue # | Dilution |
| --- | --- | --- | --- |
| Adiponectin | BD | 611644 | 1:1000 |
| Akt | Cell Signaling Technology | 4685 | 1:1000 |
| ARNTL1 (BMAL1) | Cell Signaling Technology | 14020 | 1:1000 |
| ARNTL2 (BMAL2) | Novus Biologicals | NBP2-32423 | 1:1000 |
| C/EBPβ | Santa Cruz | SC-150 | 1:1000 |
| ERK1/2 | Cell Signaling Technology | 9102 | 1:1000 |
| FABP4 | Cayman | 10004944 | 1:1000 |
| KLF4 | Cell Signaling Technology | 4038S | 1:1000 |
| P-Akt S473 | Cell Signaling Technology | 4060 | 1:1000 |
| P-Akt T308 | Cell Signaling Technology | 4056 | 1:1000 |
| P-ERK1/2 | Cell Signaling Technology | 9101 | 1:1000 |
| PPARγ | Cell Signaling Technology | 2435 | 1:1000 |
| P-S6K | Cell Signaling Technology | 9234 | 1:1000 |
| S6K | Cell Signaling Technology | 9202 | 1:1000 |
| V5 Tag | Invitrogen | R960-25 | 1:1000 |
| β-Actin | Sigma Aldrich | A5441 | 1:100.000 |
