# Supplementary material for: The circadian transcription factor ARNTL2 is regulated by weight-loss interventions in human white adipose tissue and inhibits adipogenesis
Source: Cell Death Discov. 2022 Nov 3;8:443. doi: 10.1038/s41420-022-01239-3 (PMC9633602; doi:10.1038/s41420-022-01239-3)
Supplement: Supplementary file 6 — Supplementary Table 3 [file 41420_2022_1239_MOESM6_ESM.docx]

**Supplementary Table 3:** Primer sequences for quantitative Real-Time PCR analysis.

| Primer | Sequence 5´- 3´ | Reference |
| --- | --- | --- |
| Actin_beta_hum_for | AGAAAATCTGGCACC ACACC | ([1](#_ENREF_1)) |
| Actin_beta_hum_rev | AGAGGCGTACAGGGATAGCA | ([1](#_ENREF_1)) |
| AdipoQ_hum_for | CCTGGTGAGAAGGGTGAGAA | ([2](#_ENREF_2)) |
| AdipoQ_hum_rev | GTAAAGCGAATGGGCATGTT | ([2](#_ENREF_2)) |
| Ap2-hum-II_for | TACTGGGCCAGGAATTTGAC | ([3](#_ENREF_3)) |
| Ap2-hum-II_rev | GTGGAAGTGACGCCTTTCAT | ([3](#_ENREF_3)) |
| ARNTL1_hum_for | TGCCACCAATCCATACACAG | ([4](#_ENREF_4)) |
| ARNTL1_hum_rev | TTCCCTCGGTCACATCCTAC | ([4](#_ENREF_4)) |
| ARNTL2_hum_for | GCTAGAGGCTACCAGGCAAAACC | ([5](#_ENREF_5)) |
| ARNTL2_hum_rev | GGTCCACTGGATGTCACTGAAGTC | ([5](#_ENREF_5)) |
| hC/EBPb_for | AAGCACAGCGACGAGTACAA | ([3](#_ENREF_3)) |
| hC/EBPb_rev | AGCTGCTCCACCTTCTTCTG | ([3](#_ENREF_3)) |
| KLF15_hum_for | CAAAAGCAGCCACCTCAAG | ([6](#_ENREF_6)) |
| KLF15_hum_rev | GTCAGAGCGCGAGAACCT | ([6](#_ENREF_6)) |
| KLF4_hum_for | GAAATTCGCCCGCTCCGATGA | ([7](#_ENREF_7)) |
| KLF4_hum_rev | CTGTGTGTTTGCGGTAGTGCC | ([7](#_ENREF_7)) |
| PPARg2_hum_for | ATGGGTGAAACTCTGGGAGA | ([8](#_ENREF_8)) |
| PPARg2_hum_rev | TGGAATGTCTTCGTAATGTGGA | ([8](#_ENREF_8)) |

**Supplementary References**

1. Ejaz A, Mitterberger MC, Lu Z, Mattesich M, Zwierzina ME, Horl S, et al. Weight Loss Upregulates the Small GTPase DIRAS3 in Human White Adipose Progenitor Cells, Which Negatively Regulates Adipogenesis and Activates Autophagy via Akt-mTOR Inhibition. EBioMedicine. 2016;6:149-61.

2. Mitterberger MC, Mattesich M, Zwerschke W. Bariatric surgery and diet-induced long-term caloric restriction protect subcutaneous adipose-derived stromal/progenitor cells and prolong their life span in formerly obese humans. Experimental gerontology. 2014;56:106-13.

3. Lechner S, Mitterberger MC, Mattesich M, Zwerschke W. Role of C/EBPbeta-LAP and C/EBPbeta-LIP in early adipogenic differentiation of human white adipose-derived progenitors and at later stages in immature adipocytes. Differentiation; research in biological diversity. 2013;85(1-2):20-31.

4. Ao Y, Zhao Q, Yang K, Zheng G, Lv X, Su X. A role for the clock period circadian regulator 2 gene in regulating the clock gene network in human oral squamous cell carcinoma cells. Oncology letters. 2018;15(4):4185-92.

5. Olkkonen J, Kouri VP, Kuusela E, Ainola M, Nordstrom D, Eklund KK, et al. DEC2 Blocks the Effect of the ARNTL2/NPAS2 Dimer on the Expression of PER3 and DBP. Journal of circadian rhythms. 2017;15:6.

6. Matoba K, Lu Y, Zhang R, Chen ER, Sangwung P, Wang B, et al. Adipose KLF15 Controls Lipid Handling to Adapt to Nutrient Availability. Cell reports. 2017;21(11):3129-40.

7. Li J, Zheng H, Yu F, Yu T, Liu C, Huang S, et al. Deficiency of the Kruppel-like factor KLF4 correlates with increased cell proliferation and enhanced skin tumorigenesis. Carcinogenesis. 2012;33(6):1239-46.

8. Ejaz A, Mattesich M, Zwerschke W. Silencing of the small GTPase DIRAS3 induces cellular senescence in human white adipose stromal/progenitor cells. Aging. 2017;9(3):860-79.
