# Supplementary figures and images for: The circadian transcription factor ARNTL2 is regulated by weight-loss interventions in human white adipose tissue and inhibits adipogenesis
Source: Cell Death Discov. 2022 Nov 3;8:443. doi: 10.1038/s41420-022-01239-3 (PMC9633602; doi:10.1038/s41420-022-01239-3)

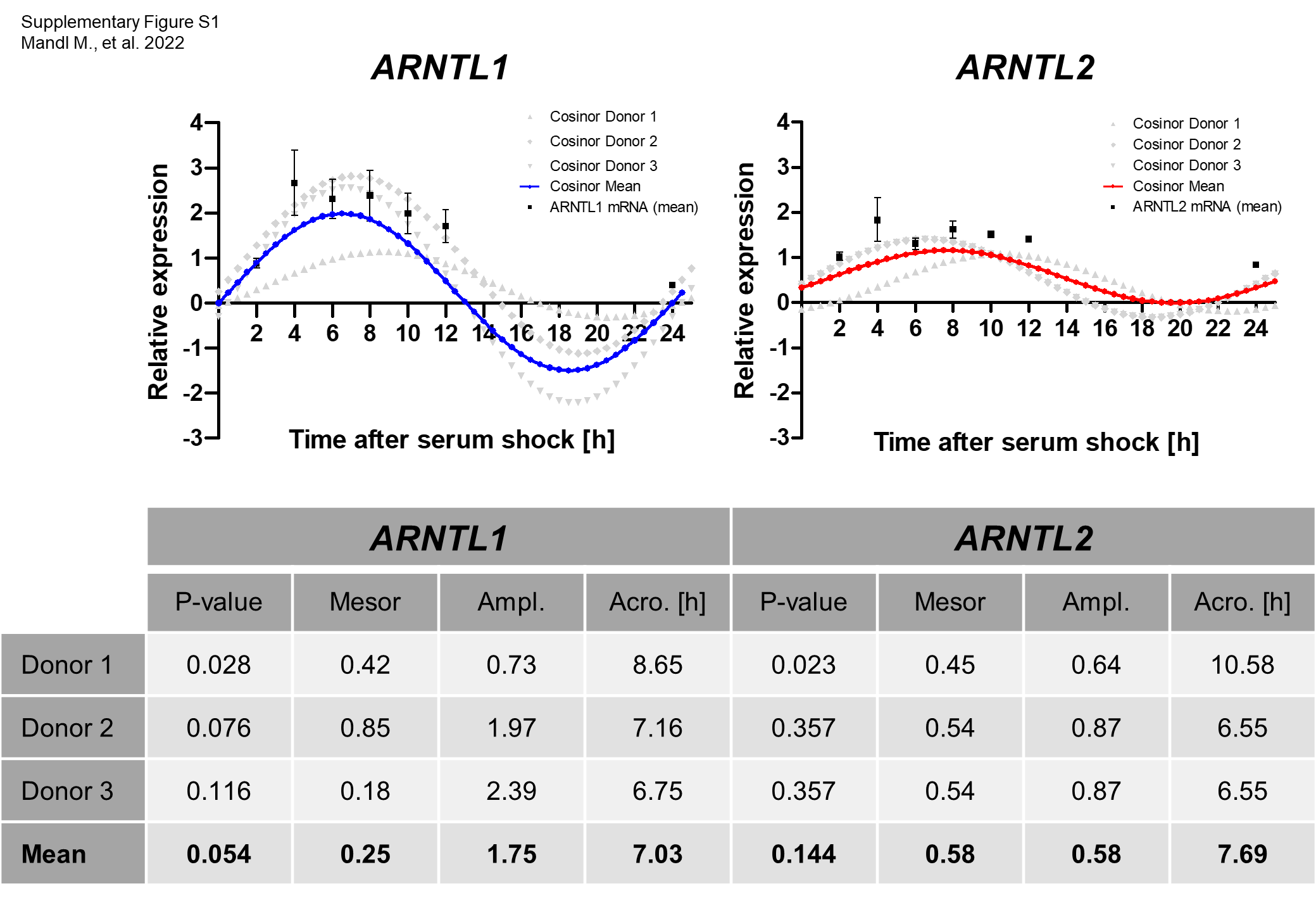

Supplement: Supplementary file 7 — Supplementary Figure S1 [file 41420_2022_1239_MOESM7_ESM.tif]

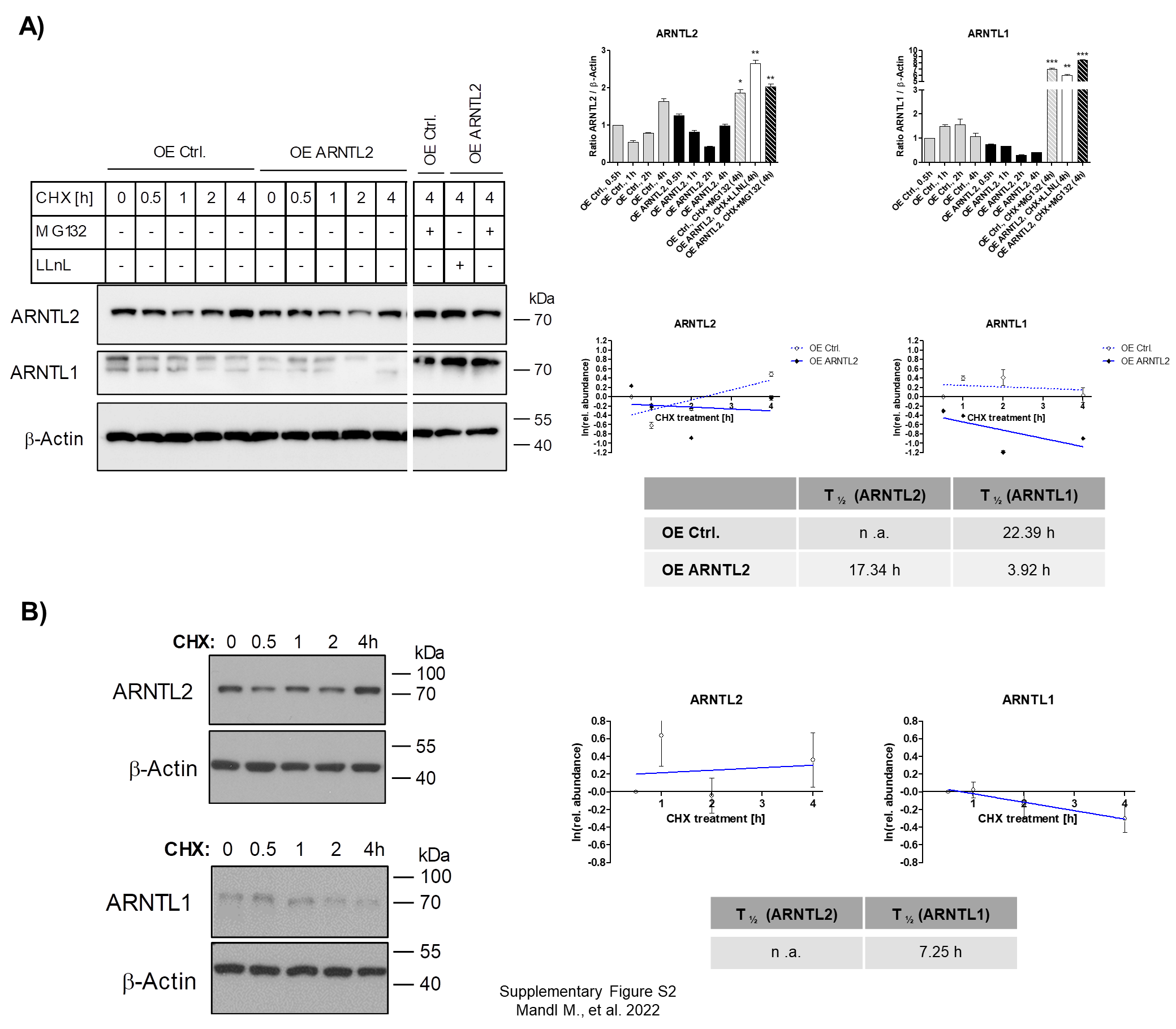

Supplement: Supplementary file 8 — Supplementary Figure S2 [file 41420_2022_1239_MOESM8_ESM.tif]

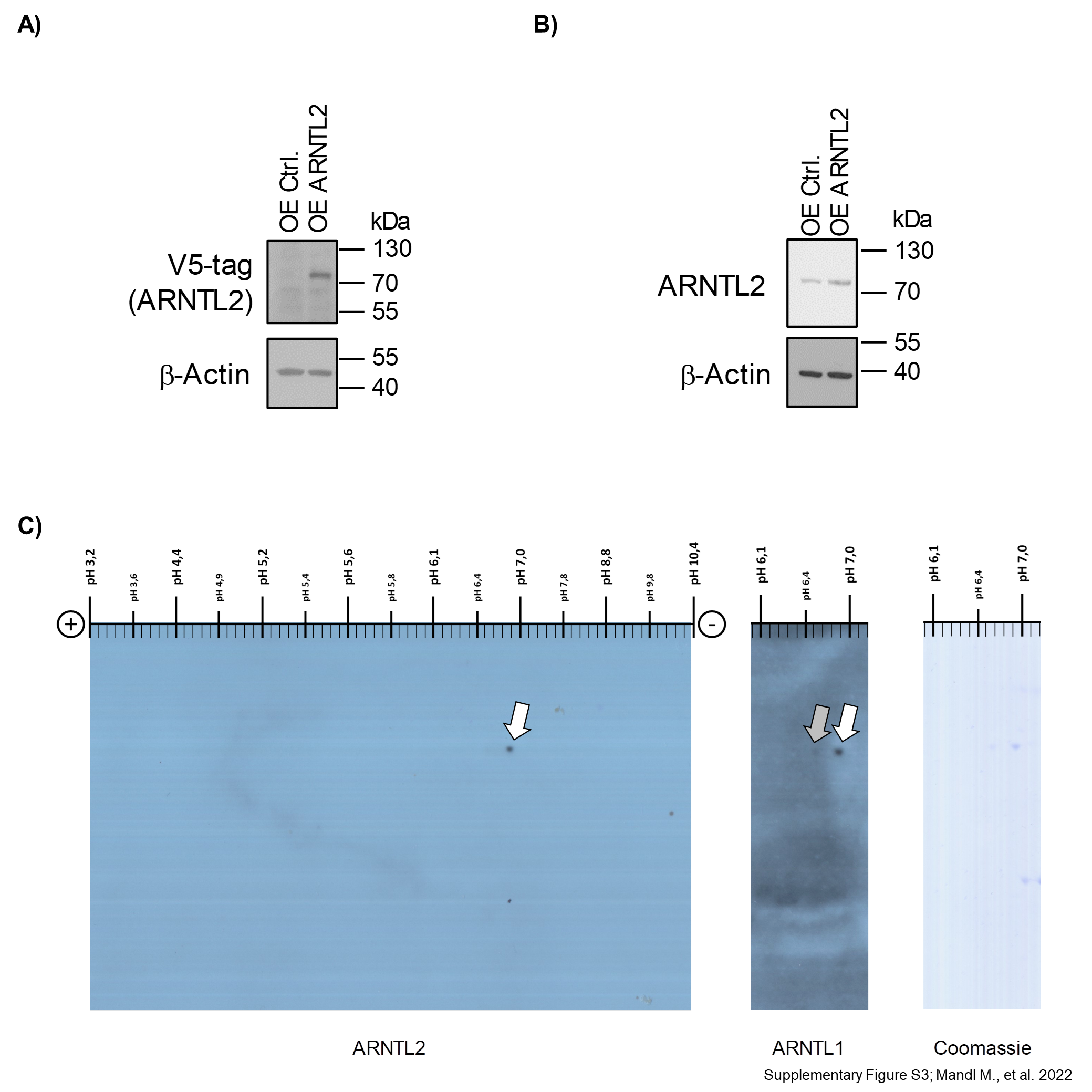

Supplement: Supplementary file 9 — Supplementary Figure S3 [file 41420_2022_1239_MOESM9_ESM.tif]
