# Supplementary material for: The circadian transcription factor ARNTL2 is regulated by weight-loss interventions in human white adipose tissue and inhibits adipogenesis
Source: Cell Death Discov. 2022 Nov 3;8:443. doi: 10.1038/s41420-022-01239-3 (PMC9633602; doi:10.1038/s41420-022-01239-3)

Original Western Blots

Figure 2B

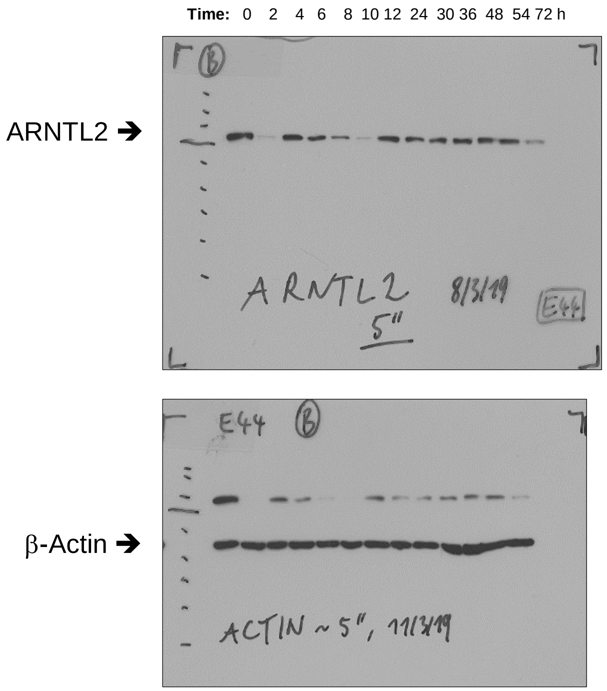
Time: 0 2 4 6 8 10 12 24 30 36 48 54 72 h
ARNTL2
β-Actin
ARNTL2
5"
8/3/19
E44
E44
ACTIN ~ 5", 11/3/19

Figure 2E

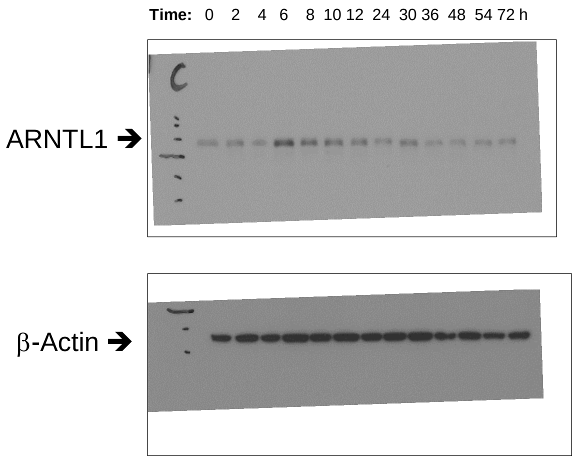

Figure 2H

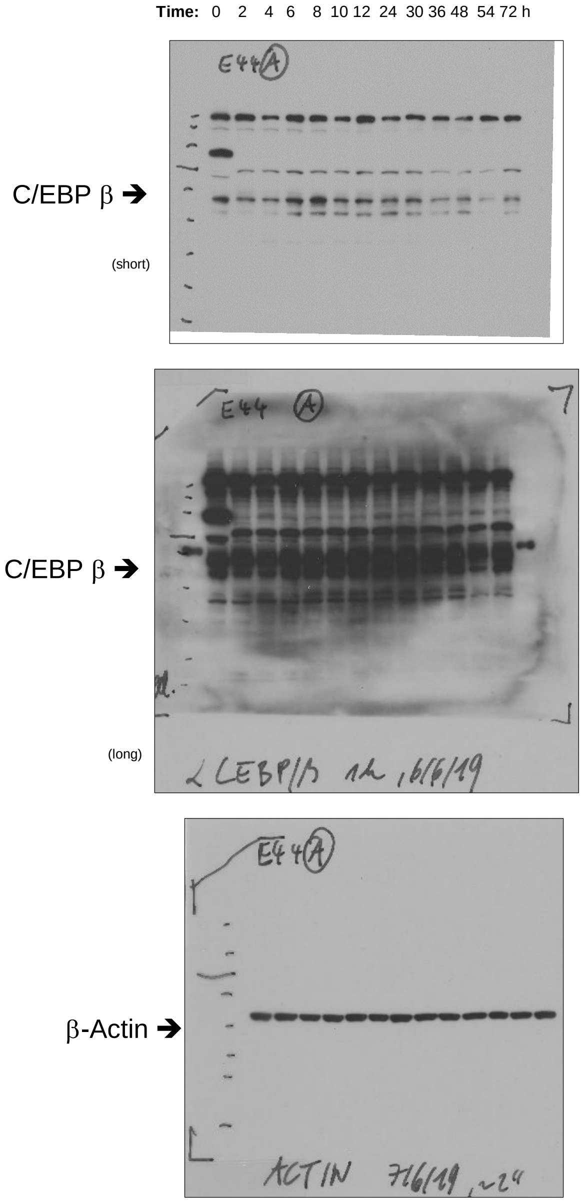
Time: 0 2 4 6 8 10 12 24 30 36 48 54 72 h
C/EBP β
(short)
C/EBP β
(long)
β-Actin

Figure 2K

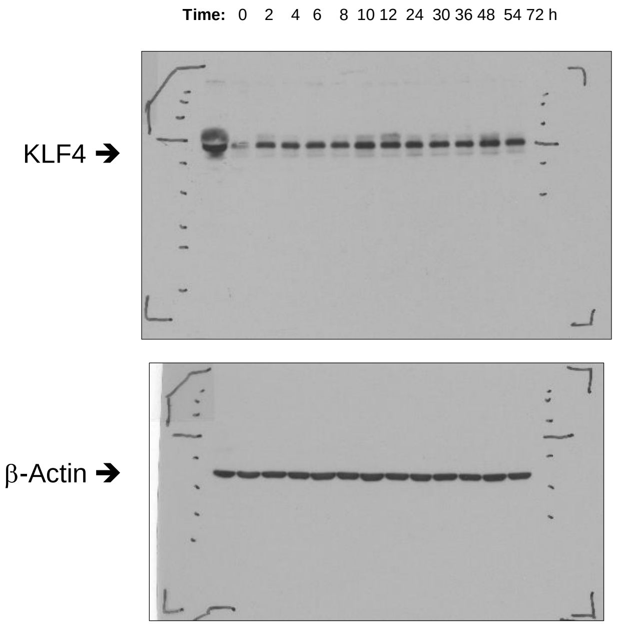

Figure 3B

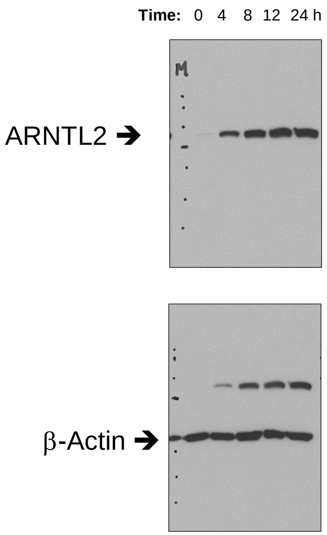

Figure 3E

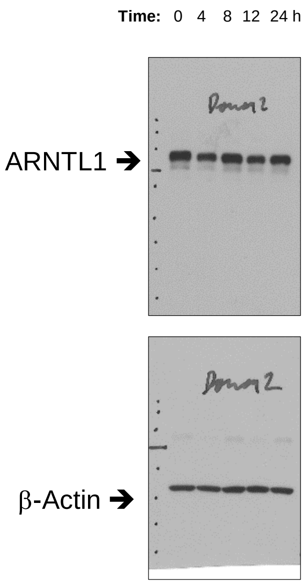

Figure 4

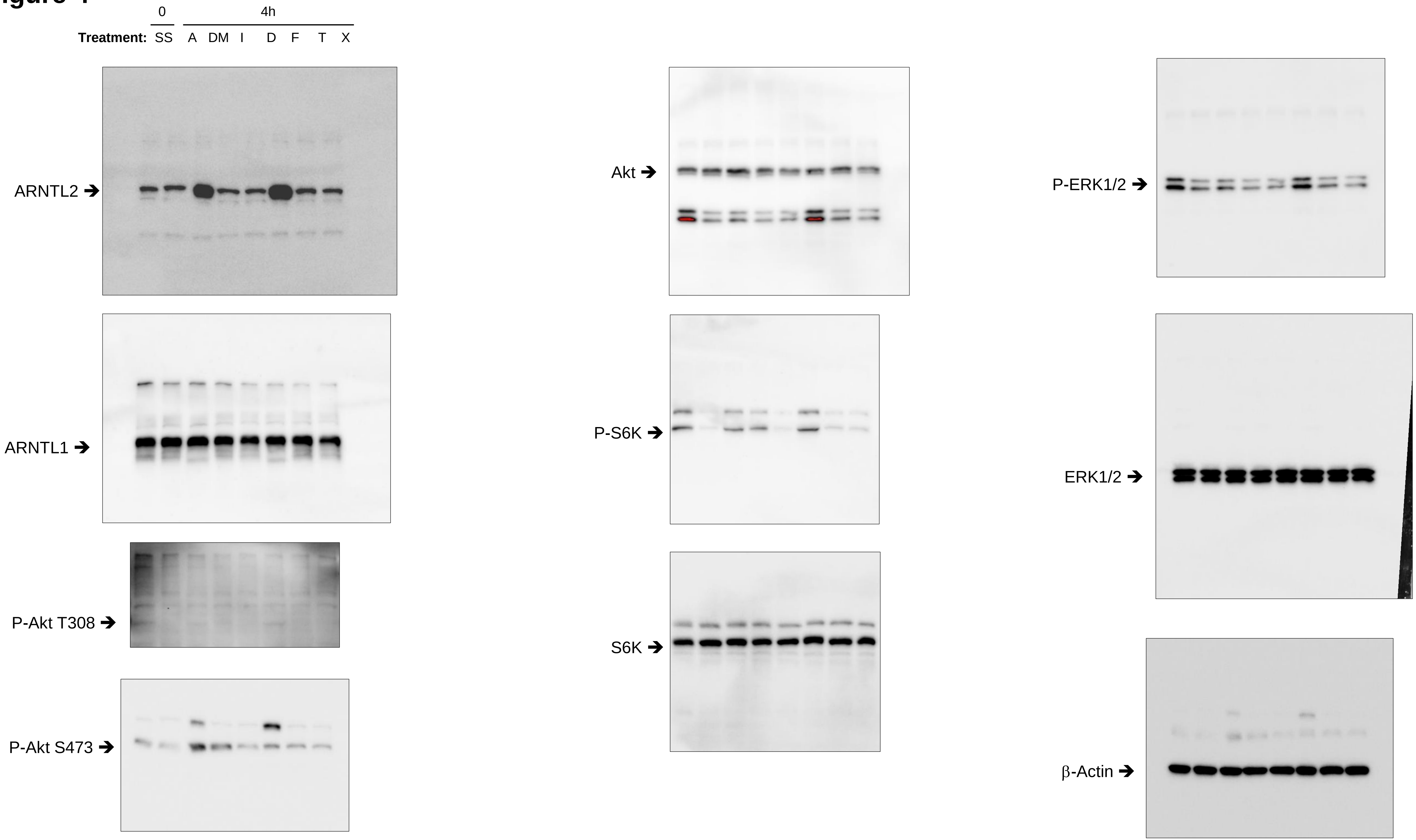

0
4h
Treatment: SS A DM I D F T X
ARNTL2
ARNTL1
P-Akt T308
P-Akt S473
Akt
P-S6K
S6K
P-ERK1/2
ERK1/2
β-Actin

Figure 5A – left panel

		DM, 30min							
DMSO	+	+							
U0126			+			+		+	+
Akt Inh.				+		+	+		+
Rapamycin					+		+	+	+

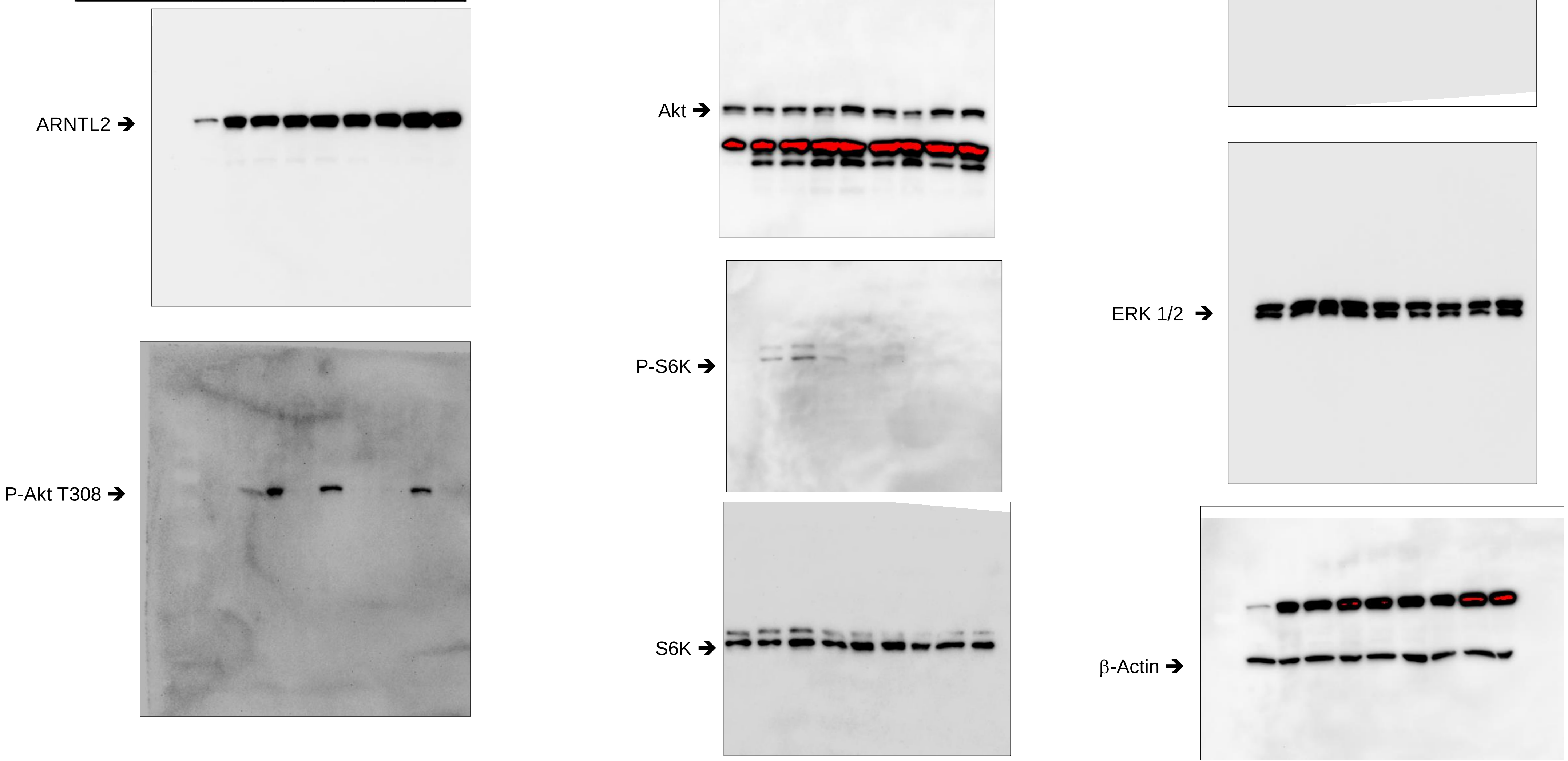

Figure 5A – right panel

		DM, 4h							
DMSO	+	+							
U0126			+			+		+	+
Akt Inh.				+		+	+		+
Rapamycin					+		+	+	+

ARNTL2 →

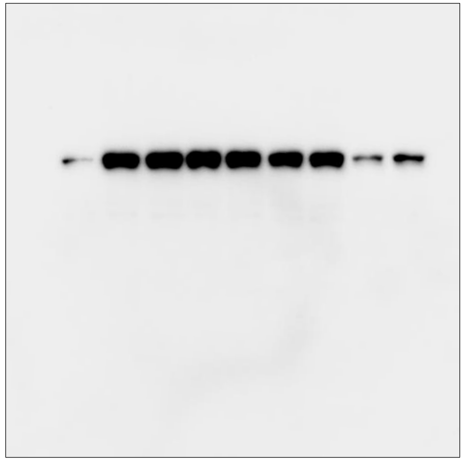

P-Akt T308 →

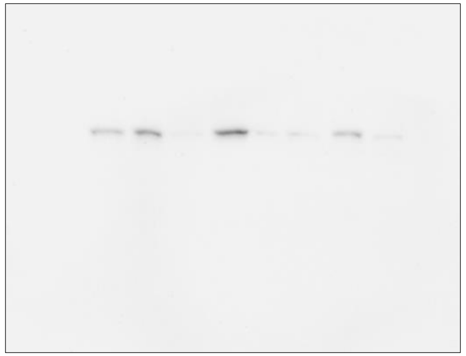

P-Akt S473 →

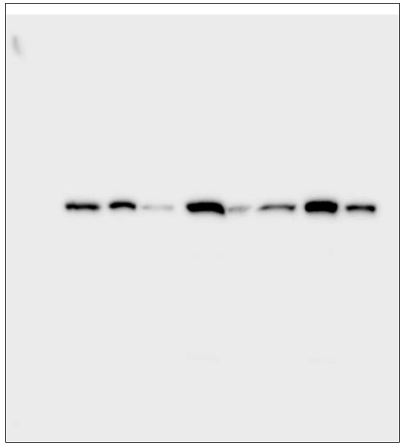

Akt →

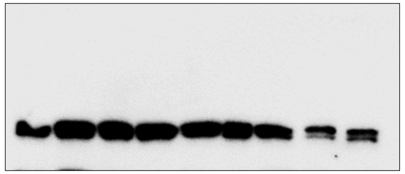

P-S6K →

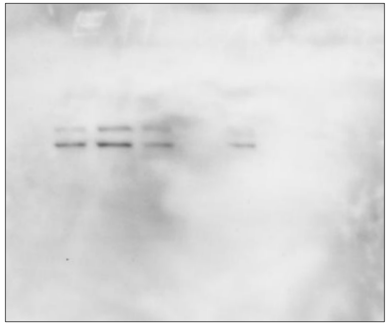

S6K →

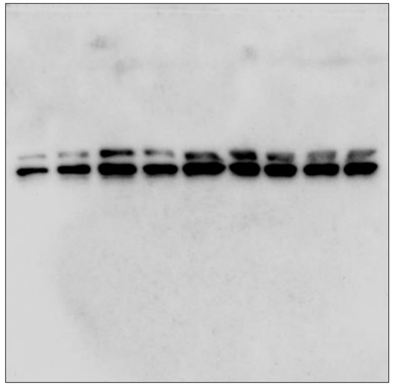

P-ERK 1/2 →

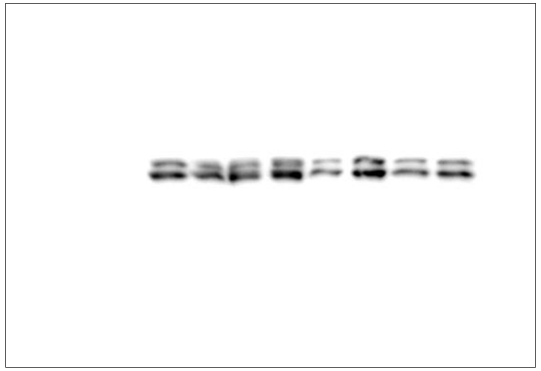

ERK 1/2 →

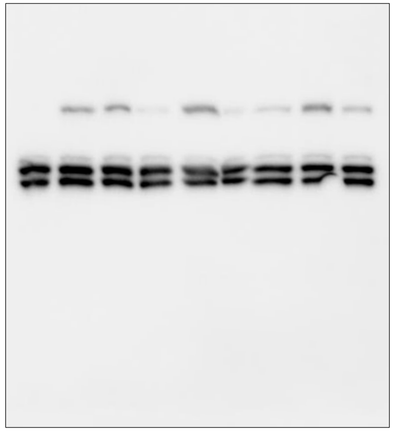

β-Actin →

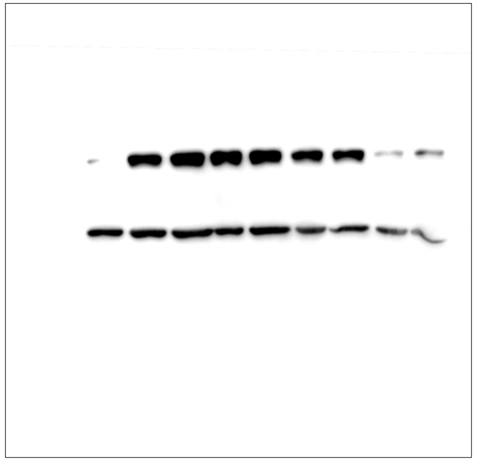

Figure 5B

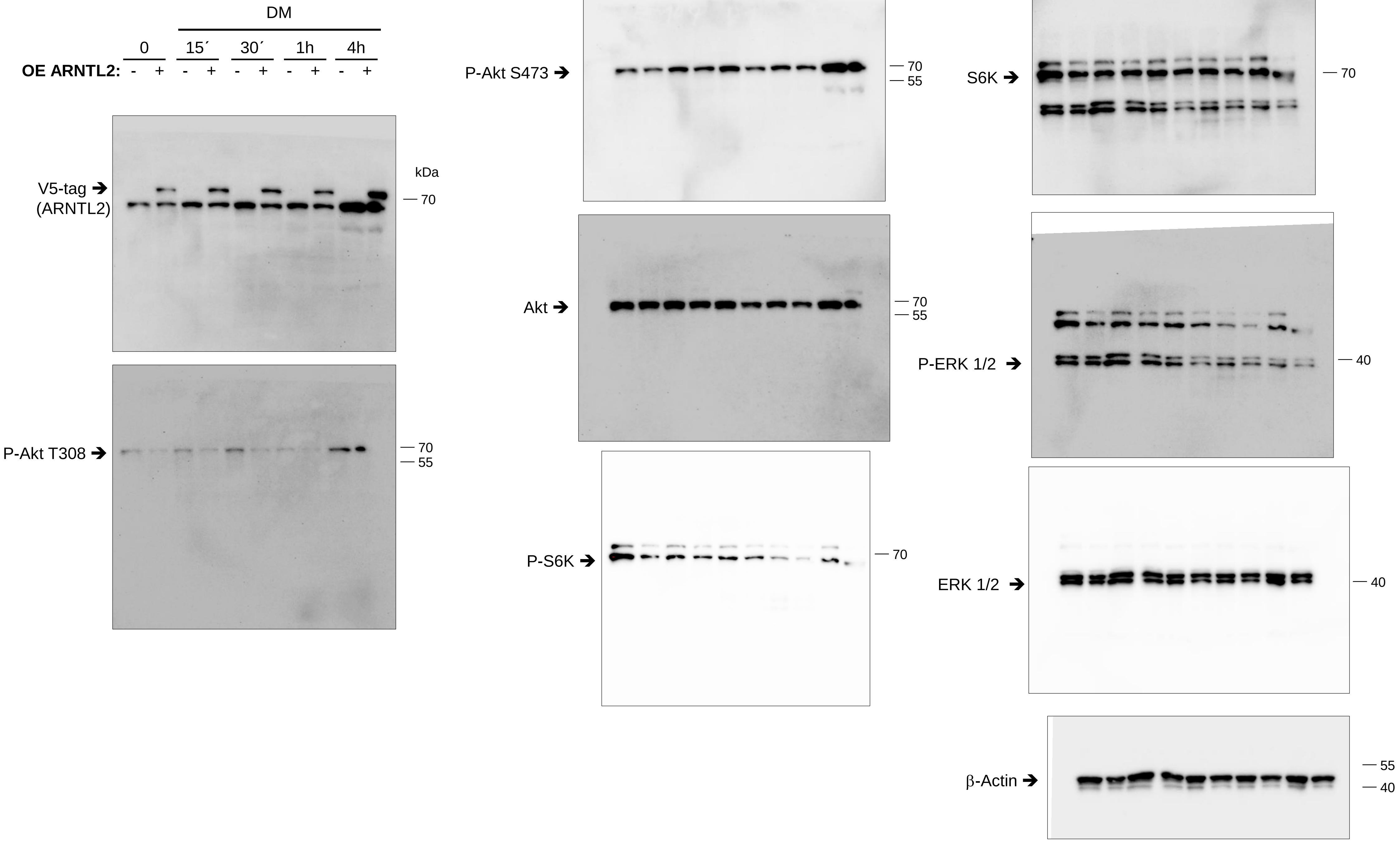
DM
0
15´
30´
1h
4h
OE ARNTL2: - + - + - + - + - +
kDa
V5-tag (ARNTL2)
70
P-Akt T308
70
55
P-Akt S473
70
55
Akt
70
55
P-S6K
70
S6K
70
P-ERK 1/2
40
ERK 1/2
40
β-Actin
55
40

Figure 6

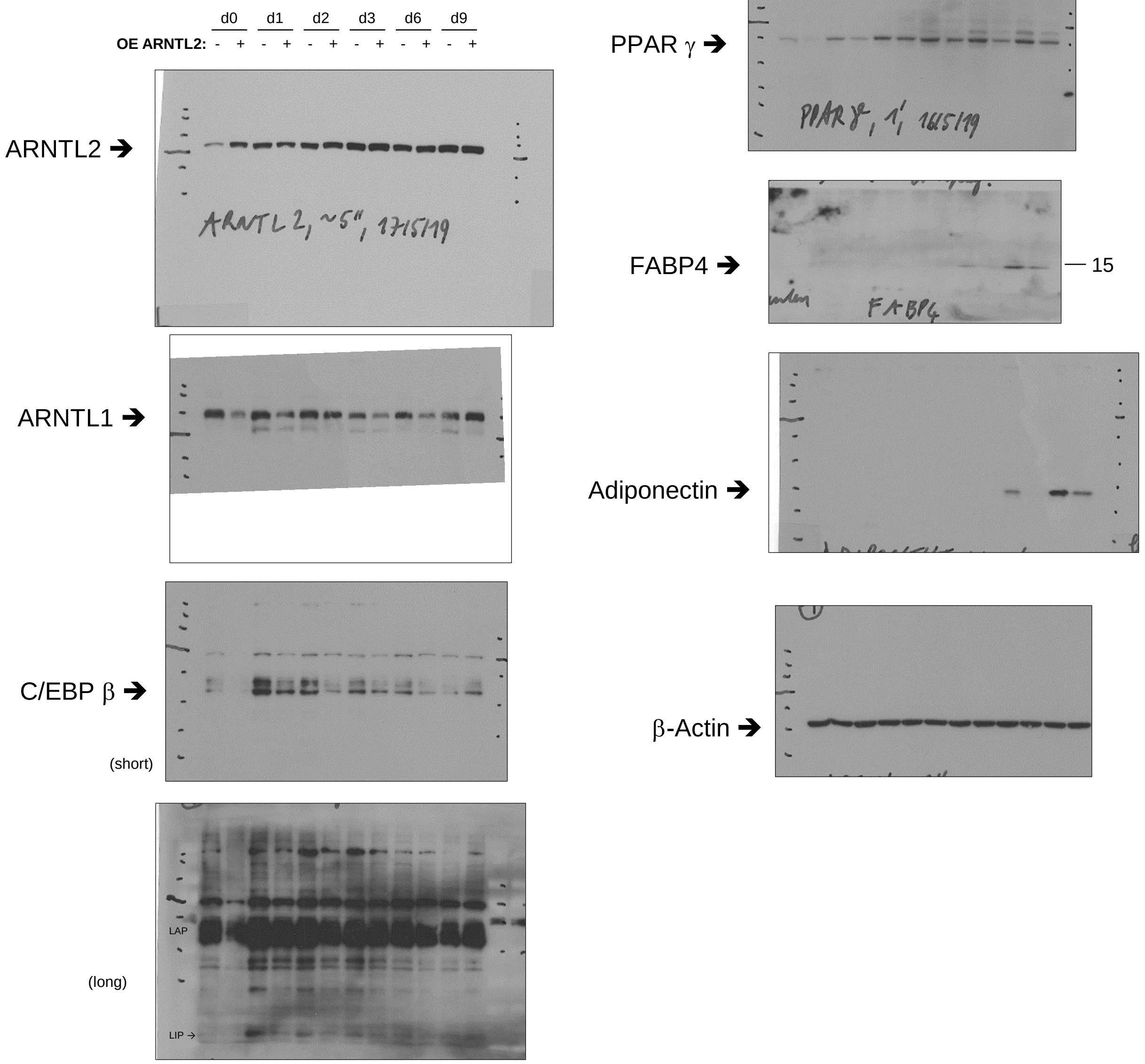
d0 d1 d2 d3 d6 d9
OE ARNTL2: - + - + - + - + - + - +
ARNTL2
ARNTL1
C/EBP β
(short)
(long)
LAP
LIP
PPAR γ
FABP4
15
Adiponectin
β-Actin

Figure 7

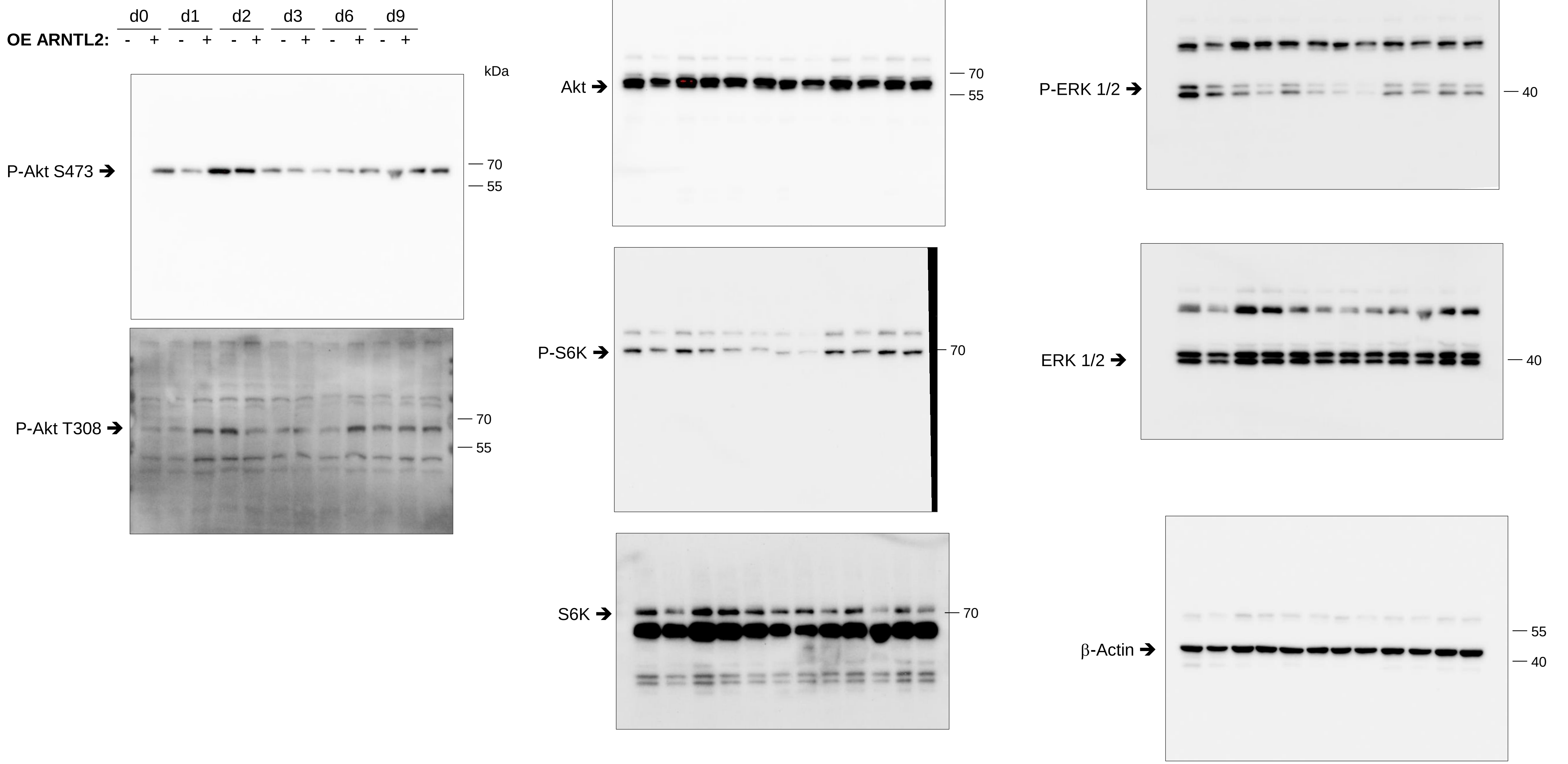
d0
d1
d2
d3
d6
d9
OE ARNTL2: - + - + - + - + - + - +
kDa
P-Akt S473
70
55
P-Akt T308
70
55
Akt
70
55
P-S6K
70
S6K
70
P-ERK 1/2
40
ERK 1/2
40
β-Actin
55
40

Figure 8a

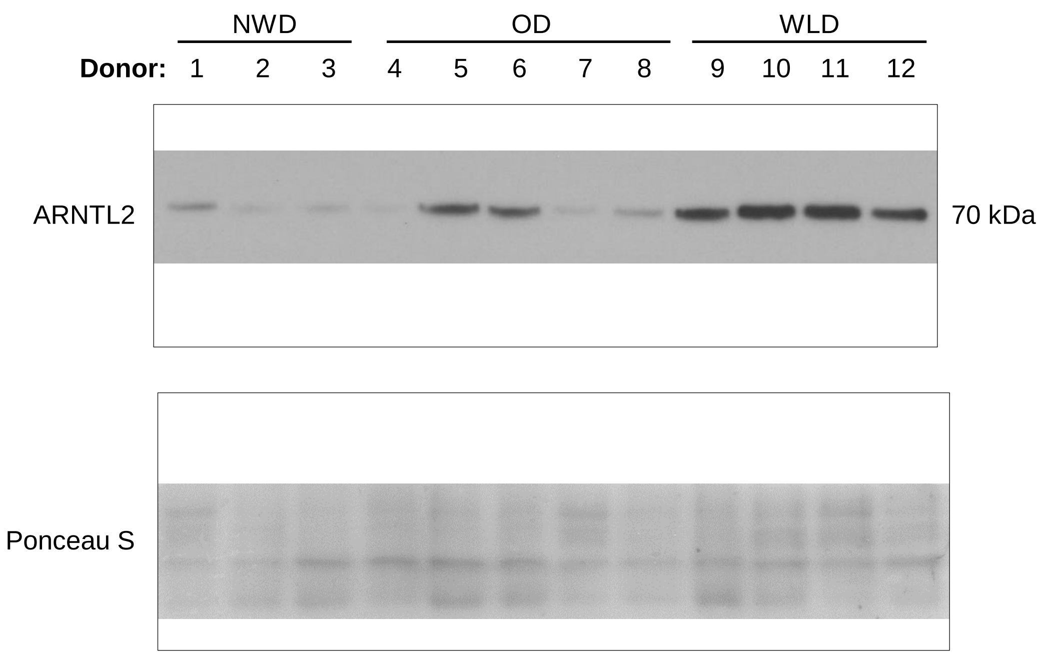

Figure 8b

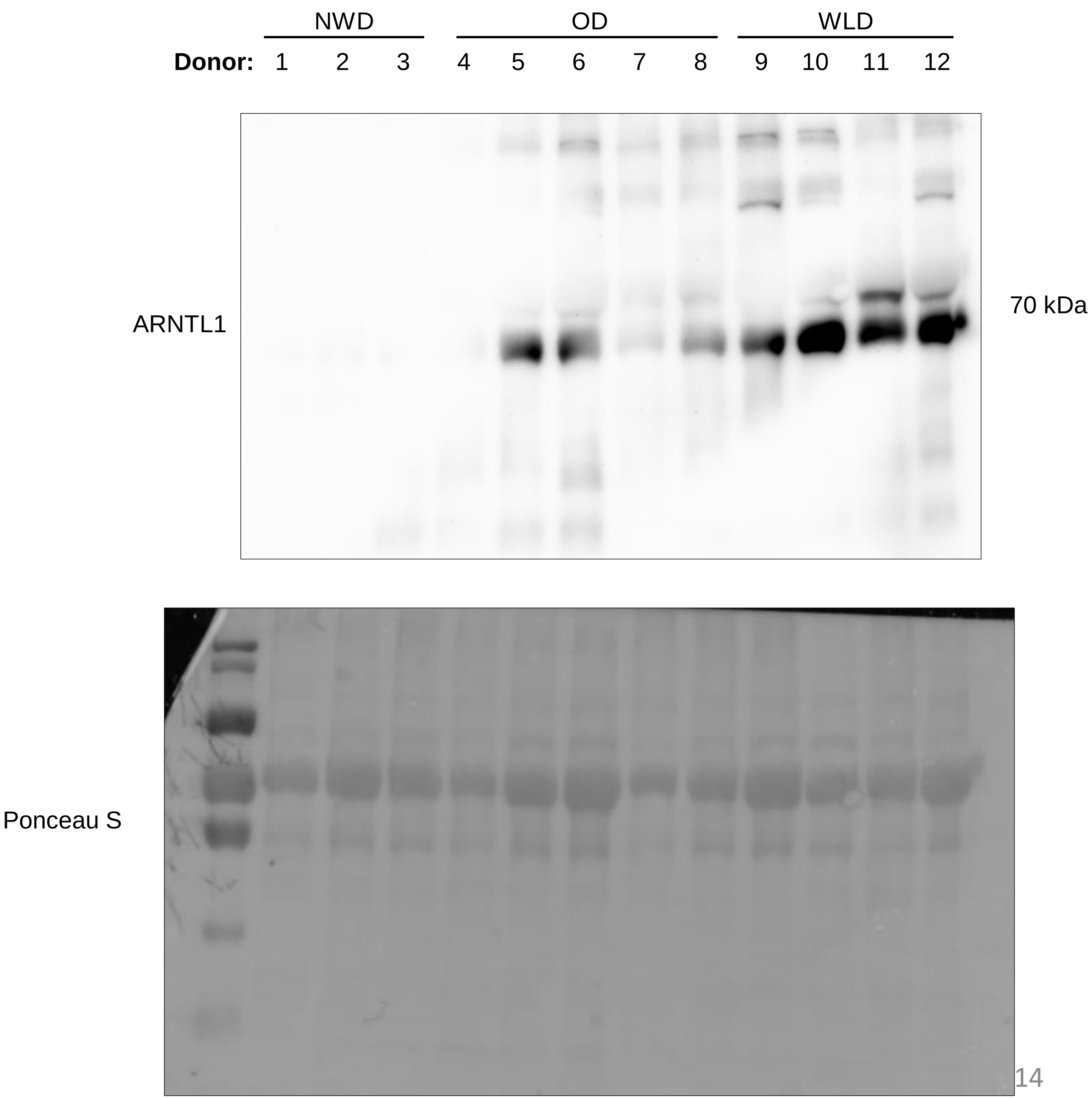

Supplementary Figure S2 A

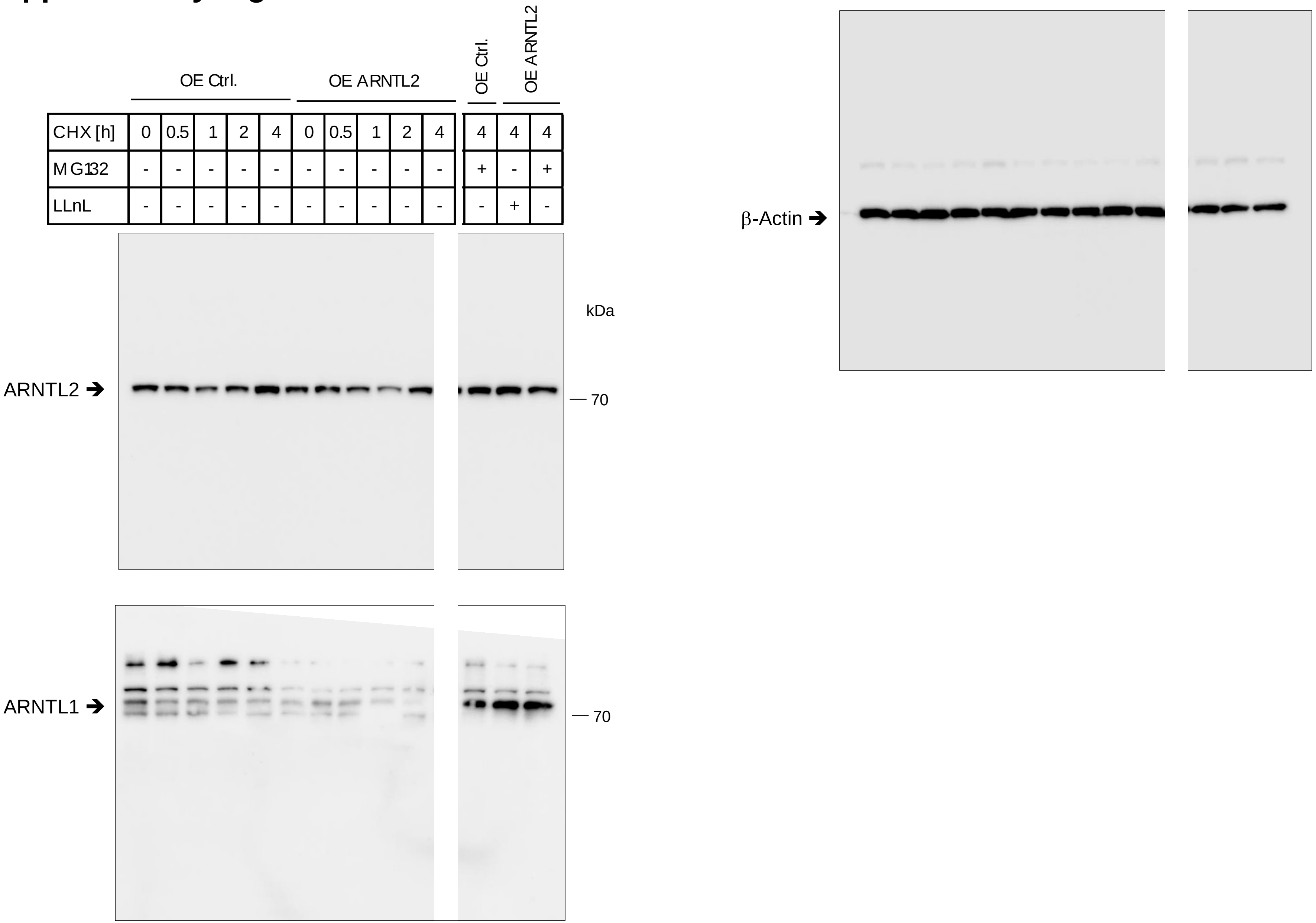

Supplementary Figure S2 B

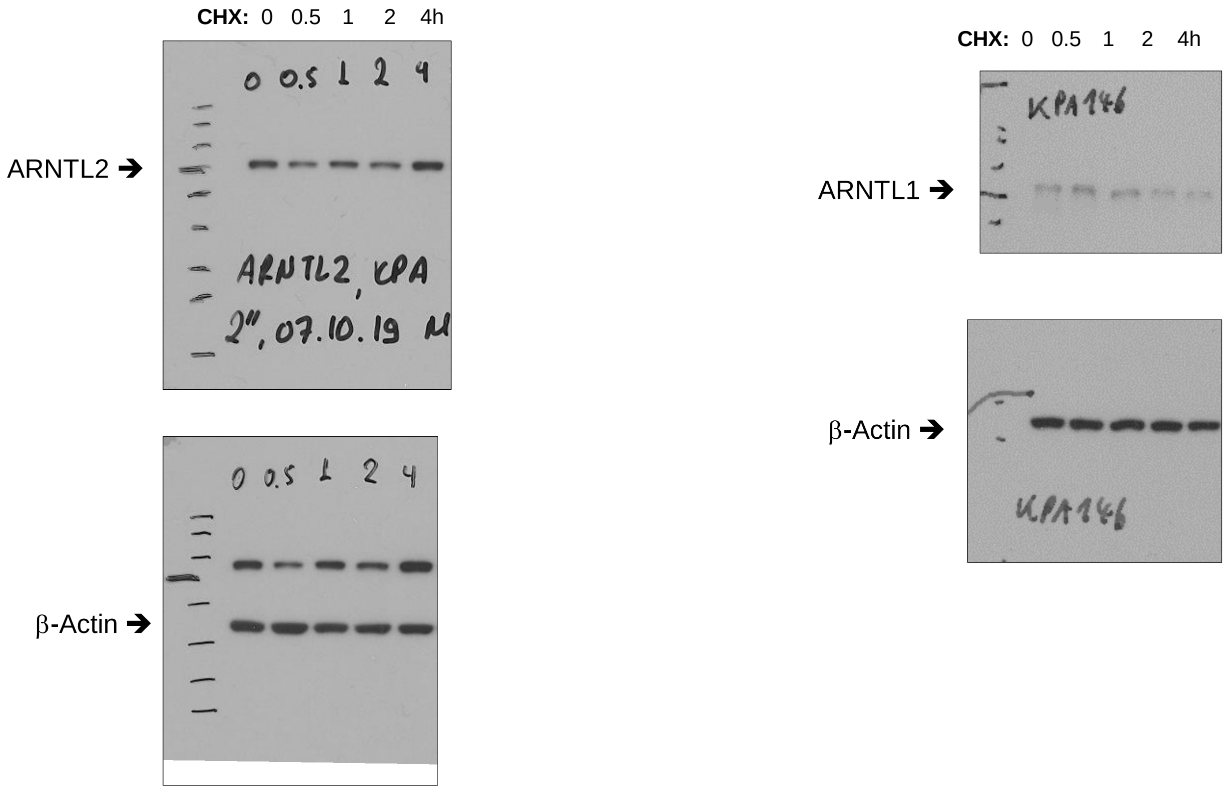

Supplementary Figure S3 A

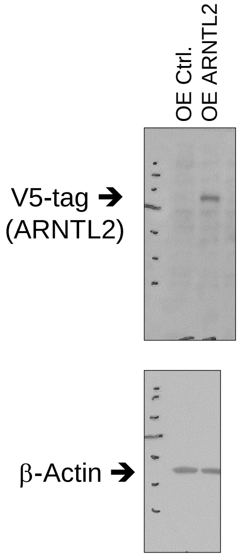

Supplementary Figure S3 B

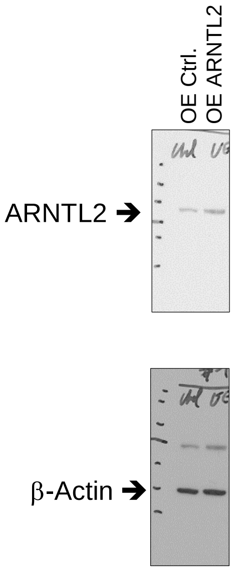

Supplement: Supplementary file 10 — Original Data File [file 41420_2022_1239_MOESM10_ESM.pdf]
